# Identification of a novel compound that simultaneously impairs the ubiquitin-proteasome system and autophagy

**DOI:** 10.1080/15548627.2021.1988359

**Published:** 2021-11-05

**Authors:** Tatiana A. Giovannucci, Florian A. Salomons, Henriette Stoy, Laura K. Herzog, Shanshan Xu, Weixing Qian, Lara G. Merino, Maria E. Gierisch, Martin Haraldsson, Alf H. Lystad, Hanna Uvell, Anne Simonsen, Anna-Lena Gustavsson, Michaela Vallin, Nico P. Dantuma

**Affiliations:** a Department of Cell and Molecular Biology (CMB), Karolinska Institutet, Stockholm, Sweden; b Laboratories for Chemical Biology Umeå, Chemical Biology Consortium Sweden (CBCS), Umeå University, Umeå, Sweden; c Chemical Biology Consortium Sweden (CBCS), Science for Life Laboratory, Department of Medical Biochemistry and Biophysics, Karolinska Institutet, Stockholm, Sweden; d Department of Molecular Medicine, Institute of Basic Medical Sciences and Centre for Cancer Cell Reprogramming, Institute of Clinical Medicine, Faculty of Medicine, University of Oslo, Blindern, Oslo, Norway; e Department of Molecular Cell Biology, Institute for Cancer Research, Oslo University Hospital, Montebello, Oslo, Norway

**Keywords:** Autophagy, compound screen, inhibitor, proteostasis, stress response, ubiquitin-proteasome system

## Abstract

The ubiquitin-proteasome system (UPS) and macroautophagy/autophagy are the main proteolytic systems in eukaryotic cells for preserving protein homeostasis, i.e., proteostasis. By facilitating the timely destruction of aberrant proteins, these complementary pathways keep the intracellular environment free of inherently toxic protein aggregates. Chemical interference with the UPS or autophagy has emerged as a viable strategy for therapeutically targeting malignant cells which, owing to their hyperactive state, heavily rely on the sanitizing activity of these proteolytic systems. Here, we report on the discovery of CBK79, a novel compound that impairs both protein degradation by the UPS and autophagy. While CBK79 was identified in a high-content screen for drug-like molecules that inhibit the UPS, subsequent analysis revealed that this compound also compromises autophagic degradation of long-lived proteins. We show that CBK79 induces non-canonical lipidation of MAP1LC3B/LC3B (microtubule-associated protein 1 light chain 3 beta) that requires ATG16L1 but is independent of the ULK1 (unc-51 like autophagy activating kinase 1) and class III phosphatidylinositol 3-kinase (PtdIns3K) complexes. Thermal preconditioning of cells prevented CBK79-induced UPS impairment but failed to restore autophagy, indicating that activation of stress responses does not allow cells to bypass the inhibitory effect of CBK79 on autophagy. The identification of a small molecule that simultaneously impairs the two main proteolytic systems for protein quality control provides a starting point for the development of a novel class of proteostasis-targeting drugs.

## Introduction

Intracellular protein homeostasis, i.e., proteostasis, depends on efficient recycling of defective proteins [1]. Protein recycling must be temporally and spatially regulated, and performed in an orchestrated manner, to safeguard that only designated proteins are degraded while leaving other proteins unharmed. The ubiquitin-proteasome system (UPS) and macroautophagy (herein referred to as autophagy) are the main proteolytic systems responsible for eliminating aberrant and misfolded proteins that are intercepted by protein quality control systems.

The UPS is a complex system that involves a large number of proteins and is characterized by two key players: the small protein modifier ubiquitin, which tags proteins designated for degradation, and the proteasome, a multi-subunit proteolytic complex that executes the destruction of these proteins [2]. The compartmentalized nature of the proteasome requires proteins to be unfolded in order to be translocated into the proteolytic chamber. This requirement renders difficult-to-untangle, aggregated proteins resistant to degradation by the UPS [3]. Consequently, a failure in the timely degradation of misfolded proteins typically gives rise to the appearance of insoluble protein aggregates [4].

Autophagy, on the other hand, is less sensitive to size restrictions as it does not require unfolding of substrates [5]. This pathway comprises the capturing of intracellular constituents in autophagosomes, which are double-membrane vesicles that fuse with lysosomes, where their content is hydrolyzed. Ubiquitin-like proteins belonging to the Atg8 family play a central role in the formation and maturation of autophagosomes through their conjugation to phosphatidylethanolamine (PE) [6]. In addition to facilitating the degradation of large protein complexes, protein aggregates, or dysfunctional organelles, autophagy is also an important catabolic pathway for replenishing cellular nutrients, particularly under challenging conditions of nutrient shortage [7].

Treatment of cells with specific proteasome inhibitors results in the accumulation of misfolded proteins that precipitate in intracellular aggregates [4]. Also, malfunctioning of autophagy causes impaired clearance of aggregation-prone proteins, increasing the load of protein aggregates, which may ultimately lead to cell death [8,9]. Aggregates are actively sequestered in aggresomes, which are subcellular, membrane-less structures that serve as a deposit for aggregation-prone proteins [10,11]. By creating a physical barrier, aggresomes confine the impact of these proteins, which are inherently toxic for eukaryotic cells [12]. As such, the presence of aggresomes is indicative for insufficient protein quality control by the UPS and/or autophagy and intimately linked to loss of proteostasis and induction of proteotoxic stress.

Proteotoxic stress is a common condition in malignant cells [13]. The constant challenge of keeping the negative consequences of proteotoxic stress in check increases the sensitivity of cancer cells to drugs that interfere with protective mechanisms aimed at restoring proteostasis, such as the UPS and autophagy [14,15]. The increased load of misfolded proteins in malignant cells as well as their altered metabolic state generates a therapeutic window at which limiting the activity of these proteolytic systems has lethal consequences for cancer cells without causing substantial collateral damage to healthy, untransformed cells. Hence, in the face of constitutive proteotoxic stress, curtailing the destruction of misfolded proteins in malignant cells causes uncontrolled accumulation of aberrant proteins that pollute their intracellular environment, resulting in induction of cell death due to an unresolvable loss of proteostasis [16].

The UPS in particular has emerged as a powerful target for a new class of cancer therapeutics, resulting in the development of highly potent proteasome inhibitors that are currently used as a first-line treatment for certain hematological malignancies [17]. Inhibitors of autophagy, such as the lysosomotropic agent hydroxychloroquine, which inhibits lysosomal degradation, are also being explored in various clinical trials for their potential in cancer treatment [18]. While both the UPS and autophagy have great potential as targets for anti-cancer drugs, inhibiting only one of these systems leaves space for adaptive responses between these partially overlapping proteolytic pathways [19]. Indeed, inhibition of autophagy is accompanied by induction of the UPS [20], while UPS inhibition results in activation of protein degradation by autophagy [21]. Accordingly, stimulation of autophagy makes cells less sensitive to the toxic effect of UPS inhibition [22], and combined inhibition of the UPS and autophagy increases induction of apoptosis in cancer cells as compared to targeting only one of these proteolytic systems [23].

Here, we report on a cell-based, high-content screening campaign for small molecules that inhibit the UPS. This resulted in the identification of a novel aminothiazole, CBK79, which not only caused a dramatic inhibition of the UPS but also impaired degradation of long-lived proteins by autophagy. The unique features of this compound cause a loss of proteostasis, which, despite activation of stress responses, cannot be resolved and eventually leads to cell death. Simultaneously impairing these complementary systems for eliminating aberrant proteins is expected to severely impair the ability of cells to cope with misfolded proteins, which provides a likely explanation for the activation of stress responses and pronounced toxicity observed in CBK79-treated cells. We propose that CBK79 provides an attractive active compound for the development of global disruptors of proteostasis with therapeutic potential.

## Results

### High-content screening for compounds that impair the ubiquitin-proteasome system

**Figure 1.**
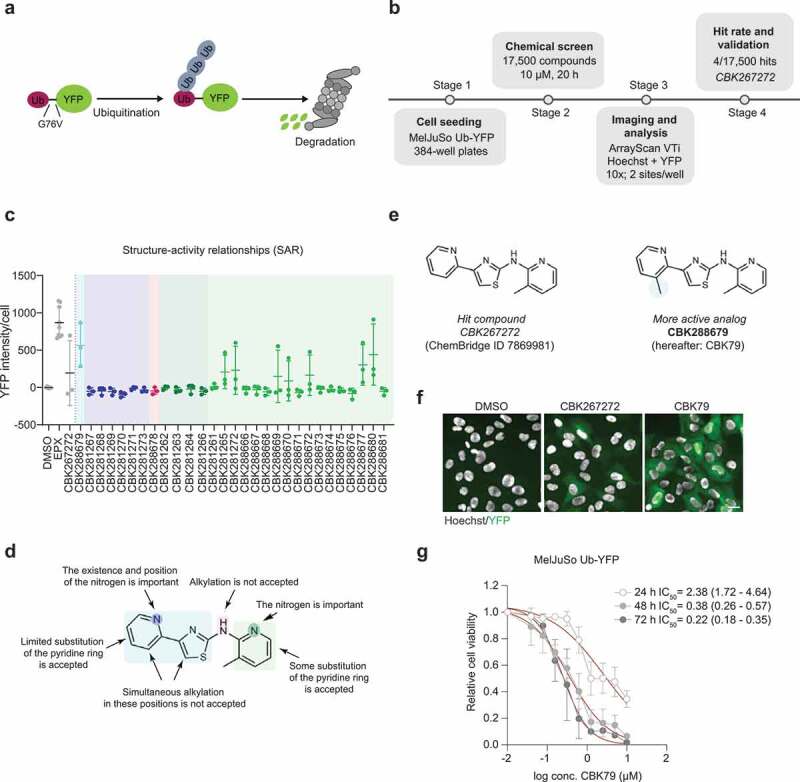
A high-content screen for inhibitors of the UPS. (A) Schematic representation of the ubiquitin-fusion degradation (UFD) signal used for the screen. MelJuSo cells stably express ubiquitin fused to YFP (Ub-YFP). A point mutation in glycine 76 to valine (G76V) disrupts the GG motif, hindering cleavage by deubiquitinating enzymes and therefore serving as a degradation signal for the UFD pathway. The rapid turnover of the protein by the proteasome provides cells with low basal YFP levels, which will be elevated upon blockade of ubiquitin-dependent degradation. (B) Workflow of the screen. 17,500 compounds were screened in an automated manner in 384-well plates. An automated analysis was performed to find compounds that elevated YFP over a predefined threshold based on the wells treated with DMSO (negative controls). CBK267272 was selected for further study. More information can be found in Fig. S1 and Table S1. (C) MelJuSo Ub-YFP cells were treated for 6 h with 29 of the structural analogues of CBK267272 at a final concentration of 20 µM. Nuclei were stained with Hoechst 33342 and cells were directly imaged live with an automated widefield microscope. Data are represented as scatter plots, where each dot represents the mean YFP nuclear intensity per cell of one experiment. The mean ± SD of three independent experiments is shown. More information can be found in Table S2. (D) Summary of the findings of the SAR. (E) Chemical structures of the initial hit compound CBK267272 and the selected optimized compound after SAR, CBK288679 (hereafter referred to as CBK79). (F) Representative maximal intensity projections of MelJuSo Ub-YFP cells treated for 6 h with CBK267272 or CBK79 (5 µM). DMSO 0.1% was used as negative control. The nuclei were counterstained with Hoechst 33342 and cells imaged live with an automated widefield microscope. Scale bar: 20 µm. (G) Dose-response curves performed with MelJuSo Ub-YFP cells. Cell viability was assessed after the indicated timepoints. Data are represented as mean ± SD of three independent experiments (except for the 48 h timepoint, which corresponds to two independent experiments). Non-linear curve fitting is depicted in red. The half-maximal inhibitory concentration (IC_50_) upon CBK79 treatment for each timepoint is shown (95% confidence intervals [CI]).

A human melanoma cell line (MelJuSo) that stably expresses a yellow fluorescent protein (YFP)-based reporter substrate of the ubiquitin-proteasome system (UPS) was used in a semi-automated, high-content screen for small molecules that impair degradation of proteins by the UPS [24]. The YFP-based reporter substrate ubiquitin^G76V^-YFP (Ub-YFP) consists of an N-terminal ubiquitin moiety that functions as a ubiquitin fusion degradation (UFD) signal, targeting the reporter for proteasomal destruction (Figure 1A) [25]. Prior to degradation, the N-terminal ubiquitin moiety must be modified by polyubiquitin chains rendering the UFD signal strictly dependent on polyubiquitination [26]. Quantitative analysis of the levels of the reporter substrate in cells treated with experimental small molecules readily enabled identification of compounds that impair the cell’s capacity to degrade UPS substrates [27]. Compounds that directly or indirectly impair any of the critical steps required for efficient ubiquitin-dependent degradation of proteins – from the initial activation of ubiquitin until the final hydrolysis in the proteasome – will be identified as hits in this phenotypic screen.

A small-molecule screening campaign was performed using a ChemBridge compound set available at the Chemical Biology Consortium Sweden (CBCS), consisting of 17,500 compounds that have been selected to cover a large chemical space. Each 384-well plate contained 32 controls (8 positive controls: 100 nM epoxomicin; 24 negative controls: 0.1% DMSO) with the remaining wells, each containing a library compound at a final concentration of 10 µM (Figure 1B). The average z’-factor of the plates was 0.54, confirming the robustness of the screening procedure. Compounds were considered as potential hits if they caused an increase in the mean fluorescent intensity that was larger than the mean fluorescence intensity of the epoxomicin control after subtraction of 3 times its standard deviation.

From 17,500 compounds, 70 compounds showed increase YFP signal in the primary screen. The images of potential hits were visually inspected in order to distinguish compounds that caused intracellular fluorescence, consistent with accumulation of Ub-YFP, from compounds that gave rise to extracellular fluorescence, indicative for autofluorescent substances. Forty-three compounds were excluded at this point. The remaining 27 compounds were analyzed for dose-dependent effects on YFP fluorescence in both the parental MelJuSo cells and Ub-YFP MelJuSo cells. The effect on cell viability in the latter was also inferred by the effect of increasing concentrations of compound on the cell count. Out of the 27 compounds, only 4 compounds (ChemBridge IDs: 5676202, 6050076, 5128401 and 7869981) did not give a fluorescent signal in the parental cell line and increased the fluorescence intensity of the Ub-YFP-expressing MelJuSo cells, while also having a negative impact on the cell count (Table S1). These four hit substances were further validated by weighing in dry substances and making new compound stocks, which were used for analysis of dose-dependent effects on YFP fluorescence (Fig. S1).

Next, we applied several computational methods to identify and remove potentially problematic compounds from a medicinal chemistry perspective. Compound 5128401 violated the Lipinski rule of 5 [28] and was therefore excluded (Fig. S1). The remaining three hit compounds did not violate Lipinski’s rule of 5, with compound 7869981 being the only compound that passed the PAINS filter [29] and had a drug-like profile according to the REOS criteria [30]. Based on the dose-response curves and these chemical criteria, we decided therefore to focus our attention on compound 7869981: 3-methyl-N-[4-(pyridine-2-yl)-1,3thiazol-2-yl]-pyridin-2-amine (CBK267272) as this compound had a drug-like profile, had the lowest molecular weight and caused a dose-dependent increase in reporter levels that was accompanied by loss of cell viability.

To further define the chemical space of this compound, we analyzed 31 structural analogues that were used in a limited structure-activity relationship (SAR) analysis (Table S2). Repositioning of the nitrogen atoms in the two pyridine rings resulted in loss of UPS inhibition, suggesting that this feature was critical for its inhibitory activity (Figure 1(C, D); Table S2). However, small substituents were accepted in the pyridine rings, which allowed us to look for more potent analogues. From the structural analogues, we selected 3‐methyl‐N‐[4‐(3‐methylpyridin‐2‐yl)‐1,3‐thiazol‐2‐yl]pyridin‐2‐amine (CBK288679, which we will refer to as CBK79) for further characterization based on its increased potency to induce reporter accumulation and cell death (Figure 1(E, F); Table S1). The MelJuSo Ub-YFP cell line displayed IC_50_ values of 2.38 µM (95% confidence interval [CI] 1.72–4.64), 0.38 µM (95% CI 0.26–0.57) and 0.22 µM (95% CI 0.18–0.35) when analyzed 24, 48 and 72 h after administration of CBK79, respectively (Figure 1G). CBK79-inflicted cell death was caspase-independent as this was not prevented by administration of the pan-caspase inhibitor Q-VD-OPh (Fig. S2).

### CBK79 causes accumulation of ubiquitin-dependent and -independent proteasome substrates

**Figure 2.**
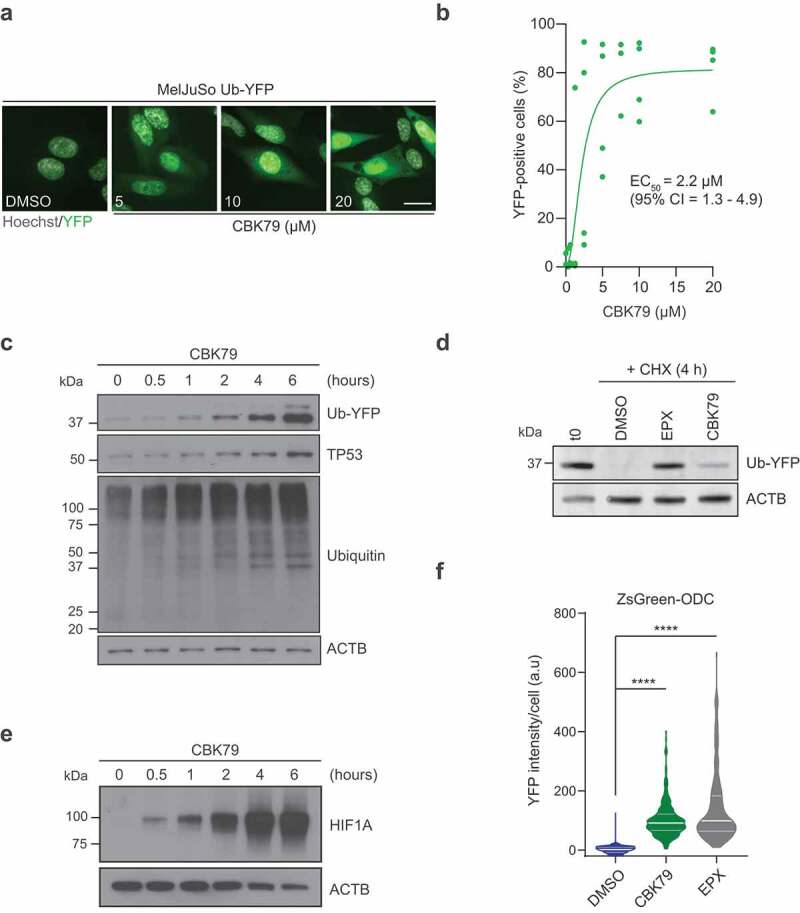
CBK79 causes accumulation of ubiquitin-dependent and -independent proteasome substrates. Representative images of MelJuSo Ub-YFP cells treated for 6 h with CBK79 at the indicated concentrations. DMSO at 0.1% was used as negative control. The nuclei were counterstained with Hoechst 33342 and cells imaged live with an automated widefield microscope. Scale bar: 20 µm. (B) Dose-response experiments performed with MelJuSo Ub-YFP cells. Cells were treated for 6 h with a range of compound concentrations. Nuclei were stained with Hoechst 33342 and cells were directly imaged live with an automated widefield microscope. Data were pooled from three independent experiments and are represented as mean ± SD. Non-linear curve fitting is depicted in green. The half-maximal effective concentration (EC_50_) upon CBK79 treatment is shown (2.2 µM, 95% confidence interval 1.3–4.9). (C) MelJuSo Ub-YFP cells were treated with CBK79 (10 µM) and harvested at the indicated timepoints. Cell lysates were analyzed by immunoblotting with the indicated antibodies. Beta-actin (ACTB) is shown as loading control. Representative blots from one of three independent experiments are shown. (D) MelJuSo Ub-YFP cells were pre-treated for 3 h with the reversible proteasome inhibitor bortezomib (25 nM) to increase the levels of YFP substrate before the treatment. Samples were taken directly after pretreatment (t0). The remaining wells were co-treated with cycloheximide (CHX, 50 µg/ml) and either DMSO 0.1%, epoxomicin (EPX, 100 nM) or CBK79 (10 µM) and harvested after 4 h (CHX 4 h). Cell lysates were analyzed by immunoblotting with the indicated antibodies. Representative blots from one of two independent experiments are shown. (E) Cell lysates from (C) were analyzed by immunoblotting with the indicated antibodies. Representative blots from one of three independent experiments are shown. (F) MelJuSo ZsGreen-ODC cells were treated for 16 h with CBK79 (2.5 µM). The proteasome inhibitor epoxomicin (100 nM) was included as positive control. Nuclei were counterstained with Hoechst 33342 and the cells imaged live with an automated widefield microscope. The nuclear YFP intensity per cell was quantified using MetaXpress. Frequency and distribution of the YFP intensity per cell after background substraction (determined as the nuclear YFP average intensity of all DMSO-treated cells) are shown as violin plots. n = 1308 cells (DMSO); n = 297 cells (CBK79) and n = 207 cells (proteasome inhibitor, epoxomicin 200 nM) from a representative experiment (of two independent experiments). Black lines within each distribution represent the median; colored lines represent the upper and lower interquartile range limits. Significant differences are based on adjusted p-values (Kruskal-Wallis [*H* = 1075, df = 2, *p* < 0.0001] with Dunn’s multiple comparisons test). ****p < 0.0001.

Administration of CBK79 to MelJuSo Ub-YFP cells resulted in a steep dose-dependent increase in YFP fluorescence that was readily detectable by fluorescence microscopy (Figure 2A). Quantitative analysis showed that the observed EC_50_ for inhibition of Ub-YFP degradation was 2.2 µM (95% CI 1.3–4.9) when assessed 6 h after administration of CBK79 (Figure 2B). An increase in Ub-YFP levels was already detectable by western blotting 1 h after administration of the compound (Figure 2C). The levels of polyubiquitinated proteins also increased with similar kinetics, indicative for a general defect in the degradation of ubiquitinated proteins in CBK79-treated cells (Figure 2C). To determine if the accumulation was caused by a delay in proteasomal degradation, we wanted to test if CBK79 inhibits the turnover of Ub-YFP. As the steady-state levels of Ub-YFP are very low and difficult to detect, we treated Ub-YFP-expressing cells briefly with the reversible proteasome inhibitor bortezomib to allow accumulation of the Ub-YFP reporter to detectable levels. After washing away the reversible proteasome inhibitor and administration of cycloheximide to switch off protein synthesis, we analyzed the clearance of the accumulated Ub-YFP in the absence or presence of CBK79. This experiment confirmed that the clearance of Ub-YFP was inhibited in the presence of CBK79, consistent with an inhibitory effect of CBK79 on proteasomal degradation (Figure 2D). Importantly, the kinetics of reporter accumulation were comparable to the increase in the levels of TP53/p53 (tumor protein p53) (Figure 2C) and HIF1A/HIF-1α (hypoxia inducible factor 1 subunit alpha) (Figure 2E), two endogenous substrates for ubiquitin-dependent proteasomal degradation [31,32]. The accumulation of endogenous substrates and ubiquitin conjugates confirms that the Ub-YFP substrate reliably reports on the overall status of the UPS.

To test whether the inhibitory effect of CBK79 is confined to ubiquitin-dependent proteasomal degradation, we took advantage of a reporter substrate that is targeted for proteasomal degradation by a motif derived from ornithine decarboxylase (ODC). The ODC degradation signal targets for proteasomal degradation without requiring polyubiquitination of the substrate [33]. Although the compound only had a marginal effect on ODC degradation under short incubations, 24 h treatment with CBK79 resulted in increased levels of the ubiquitin-independent proteasome substrate that were comparable to the levels observed in proteasome inhibitor-treated cells (median YFP levels = 2.1 a.u in DMSO *vs* 91.6 in CBK79 and 99.4 in epoxomicin) (Figure 2F; Fig. S3A). A possible explanation for the observed inhibition of ubiquitin-dependent and -independent substrates could be inhibition of the proteolytic activity of the proteasome. However, administration of CBK79 caused only a 16% decrease in the chymotrypsin-like activity of the proteasome, indicating that proteasome inhibition is not the primary cause for the accumulation of UPS substrates (Fig. S3B). Notably, it has been shown that as much as 80% reduction in chymotrypsin-like activity is required to cause a general accumulation of proteasome substrates [25,34]. Together, these data show that CBK79 causes global impairment of proteasomal degradation of ubiquitin-dependent and ubiquitin-independent substrates without substantially inhibiting the enzymatic activity of the proteasome.

### CBK79 inhibits autophagy

**Figure 3.**
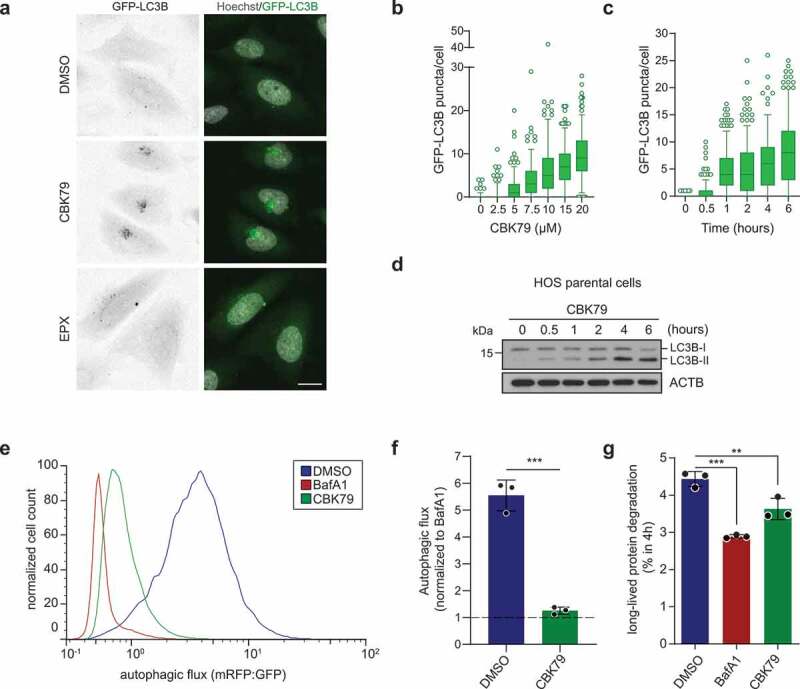
CBK79 inhibits the autophagic flux. (A) Representative images of HOS GFP-LC3B cells. Cells were treated for 4 h with DMSO 0.1%, CBK79 (10 µM) or epoxomicin (EPX, 100 nM). Nuclei were counterstained with Hoechst 33342 and the cells imaged live with an automated widefield microscope. Scale bar: 20 µm. (B) Dose-response experiments performed with HOS GFP-LC3B cells. Cells were treated for 4 h with a range of compound concentrations. Nuclei were stained with Hoechst 33342 and cells were directly imaged live with an automated widefield microscope. Single-cell measurements of GFP-LC3B puncta from a single experiment are shown (n ≥ 193 cells/condition). Data are shown as box plots with median and 5–95 percentiles. (C) HOS GFP-LC3B cells were treated with CBK79 (10 µM) for the indicated timepoints. Nuclei were stained with Hoechst 33342 and cells were directly imaged live with an automated widefield microscope. Single-cell measurements of GFP-LC3B puncta from a single experiment are shown (n > 200 cells/condition). Data are shown as box plots with median and 5–95 percentiles. (D) HOS GFP-LC3B cells were treated with CBK79 (10 µM) and harvested at the indicated timepoints. Cell lysates were analyzed by immunoblotting with the indicated antibodies. Beta-actin (ACTB) is shown as loading control. Representative blots from one out of >3 independent experiments are shown. (E) Analysis of autophagic flux in U2OS mRFP-GFP-LC3B cells treated with DMSO 0.1%, bafilomycin A_1_ (BafA1, 100 nM) or CBK79 (10 µM) for 4 h, washed briefly in saponin (0.05%) and analyzed by flow cytometry. Autophagic flux was determined as the ratio of mean mRFP- and mean GFP-fluorescence. A representative histogram from one of three independent experiments is shown. (F) Data from (E) were normalized to BafA1. Data are presented as the mean ± SD of three independent experiments (unpaired, two-tailed t-test, *t*(4) = 12.60, *p* < 0.0002). (G) Long-lived protein degradation assay in U2OS cells treated with DMSO 0.1%, bafilomycin A_1_ (BafA1, 100 nM) or CBK79 (10 µM) for 4 h. The percentage of long-lived protein degradation was quantified. Data are presented as the mean ± SD of three independent experiments, each performed in technical duplicates. Significant differences are based on adjusted p-values of multiple comparisons against DMSO (one-way ANOVA [*F*_2,6_ = 42.74, *p* = 0.0003] with Dunnett’s multiple comparisons test). **p = 0.0052; ***p = 0.0002.

In subsequent analysis of the cellular response to CBK79, we found that administration of CBK79 to human osteosarcoma (HOS) cells expressing green fluorescent protein (GFP)-tagged MAP1LC3B/LC3B (microtubule-associated protein 1 light chain 3 beta) caused the formation of cytosolic GFP-LC3B foci, which is indicative for an effect on autophagy (Figure 3A). The formation of LC3B foci was dose-dependent (Figure 3B) and time-dependent (Figure 3C) and coincided with the generation of lipidated LC3B (LC3B-II) (Figure 3D). An increase in LC3B-II can be caused by either an induction or inhibition of autophagy [35]. To distinguish between these two possibilities, we measured the autophagic flux by flow cytometry using a U2OS cell line that stably expresses LC3B carrying a tandem fluorescent tag consisting of monomeric red fluorescent protein (mRFP) and GFP. Because GFP fluorescence is quenched in the acidic lysosomal compartment, in contrast to mRFP, the mRFP:GFP ratio can be used as a readout for the autophagic flux [35]. Notably, CBK79 caused a 4.4-fold reduction in the autophagic flux that was comparable to the reduction observed after administration of the autophagy inhibitor bafilomycin A_1_ (BafA1) (Figure 3(E, F)), which blocks autophagy by inhibiting the vacuolar-type H^+^-translocating ATPase (V-ATPase) [35]. Analysis of the degradation of long-lived proteins, which are primarily degraded by autophagy, confirmed a significant inhibition in the turnover of this pool of proteins in CBK79-treated cells although to a lesser extent than observed with BafA1 administration (Figure 3G). We conclude that CBK79 not only impairs the UPS but also inhibits degradation of long-lived proteins by autophagy.

### CBK79 induces non-canonical lipidation of LC3B

**Figure 4.**
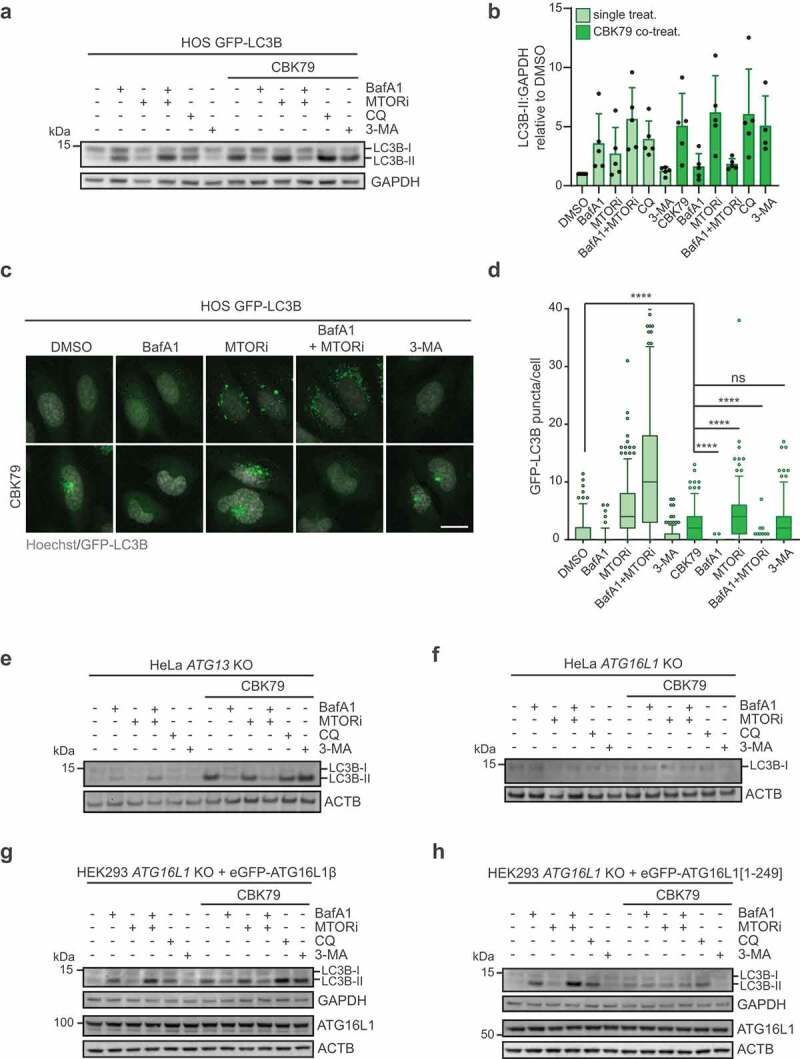
CBK79 induces non-canonical lipidation of LC3B. (A) HOS GFP-LC3B cells were treated with either DMSO 0.1% or CBK79 (10 µM) in co-treatment with the indicated autophagy modulators for 4 h. Cell lysates were analyzed by immunoblotting with the indicated antibodies. Representative blots from one of five independent experiments are shown. MTOR inhibitor (“MTORi”, Torin-1); CQ = chloroquine; 3-MA: 3-methyladenine. (B) Band intensities were measured using ImageJ. LC3B-II band was normalized to the loading control (GAPDH) and the levels are displayed relative to DMSO. Data are shown as average ± SD of five independent experiments. (C) Representative images from HOS GFP-LC3B cells treated with the indicated compounds for 4 h. Nuclei were stained with Hoechst 33342 and cells were directly imaged live with an automated widefield microscope. Scale bar: 20 µm. (D) Quantification of the GFP-LC3B puncta per cell from (**C**). Data are shown as box plots with median and 5–95 percentiles (n > 200 cells/condition). Significant differences are based on adjusted p-values between relevant conditions (Kruskal-Wallis [*H* = 1256, df = 9, *p* < 0.0001] with Dunn’s multiple comparisons test). ns > 0.9999; ****p < 0.0001. (E) HeLa *ATG13* knockout (*ATG13* KO) cells, or (F) HeLa *ATG16L1* knockout (*ATG16L1* KO) cells were treated with either DMSO 0.1% or CBK79 (10 µM) in co-treatment with the indicated autophagy modulators for 4 h. Cell lysates were analyzed by immunoblotting with the indicated antibodies. Beta-actin (ACTB) is shown as loading control. Representative blots from one of three independent experiments are shown. (G) HEK293 *ATG16L1* knockout cells stably rescued with eGFP-tagged full length ATG16L1β were treated with either DMSO 0.1% or CBK79 (5 µM) in co-treatment with the indicated autophagy modulators for 3 h. Cell lysates were analyzed by immunoblotting with the indicated antibodies. Representative blots from one of three independent experiments are shown. (H) HEK293 *ATG16L1* knockout cells stably rescued with eGFP-tagged ATG16L1[1–249] were treated with either DMSO 0.1% or CBK79 (5 µM) in co-treatment with the indicated autophagy modulators for 3 h. Cell lysates were analyzed by immunoblotting with the indicated antibodies. Representative blots from one of three independent experiments are shown.

Since the autophagic flux measured by the tandem-tagged LC3B was more severely impaired than the degradation of long-lived proteins, we had a closer look at the status of LC3B in CBK79-treated cells. As expected, co-treatment of CBK79 with MTOR (mechanistic target of rapamycin kinase) inhibitor (KU-0063794), which induces autophagy, caused an additional increase in the levels of lipidated LC3B, consistent with CBK79 obstructing the delivery of LC3B to the lysosomal compartment (Figure 4(A, B)). Accordingly, co-treatment with MTOR inhibitor also further increased the number of GFP-LC3B puncta (Figure 4(C, D)). Surprisingly, whereas treatment of cells with BafA1 caused an accumulation of lipidated LC3B that was comparable to the effect of CBK79, co-treatment of CBK79 with BafA1 prevented the appearance of LC3B-II and the formation of LC3B puncta (Figure 4(A-D)). However, administration of chloroquine (CQ), a weak base that inhibits autophagy by neutralizing the acidic lysosomal pH [35], did not prevent lipidation of LC3B, suggesting that the effect of BafA1 is not caused by preventing acidification of the lysosomes but likely relates to its ability to act as a V-ATPase inhibitor (Figure 4(A, B)).

Lipidation of LC3 that is BafA1 sensitive has also been reported for other inhibitors of autophagy and has been attributed to induction of a non-canonical pathway for lipidation of LC3 [36,37]. Canonical lipidation of LC3 to double-membrane phagophores requires the ULK1 (unc-51 like autophagy activating kinase 1) complex and the class III phosphatidylinositol 3-kinase (PtdIns3K) complex I that produces phosphatidylinositol-3-phosphate (PtdIns3P). The PtdIns3P-binding protein WIPI2B (WD repeat domain, phosphoinositide interacting 2 beta) further recruits the ATG12–ATG5-ATG16L1 complex, which functions as an E3 ligase that determines the site of LC3 lipidation [5]. In contrast, the ULK1 and PtdIns3K complexes are dispensable for the non-canonical pathway of LC3 lipidation [38].

To explore if CBK79 induces non-canonical LC3 lipidation, cells were co-treated with CBK79 and 3-methyladenine (3-MA), a compound that blocks canonical lipidation of LC3 by inhibiting the PtdIns3K complex [39]. 3-MA did not inhibit CBK79-induced LC3B lipidation and formation of GFP-LC3B puncta in HOS (Figure 4(A-D)) and HeLa cells (Fig. S4A, B), indicating that CBK79 induces LC3B lipidation in a PtdIns3P-independent, BafA1-sensitive manner, characteristic for non-canonical lipidation. Consistently, an increase in lipidated LC3B and LC3B puncta was also observed upon CBK79 treatment of HeLa cells that lack the ULK1 complex component ATG13 (Figure 4E, Fig. S4C). Importantly, HeLa cells deficient for the ATG16L1α and β isoforms, and hence lacking LC3 ligase activity, did not show LC3B lipidation nor LC3B puncta formation upon CBK79 treatment, confirming that the formation of LC3B puncta and appearance of LC3B-II is due to actual lipidation of LC3B (Figure 4F, Fig. S4D).

To further explore if the effect of CBK79 on autophagy was confined to non-canonical lipidation of LC3B, we treated ATG16L1-deficient HEK293 cell lines that ectopically expressed either GFP-tagged wild-type ATG16L1 or a C-terminally truncated ATG16L1 (ATG16L1[1–249]) that can execute canonical but is deficient for non-canonical LC3 lipidation [40]. In line with non-canonical lipidation being the primary cause for the increase in LC3B-II in response to CBK79, we found that the accumulation of LC3B-II was strongly reduced in cells deficient for non-canonical autophagy (ATG16L1[1–249]) as compared to those rescued with full-length ATG16L1 (Figure 4(G, H)). A small 3-MA-sensitive increase in lipidated LC3B was still observed upon CBK79 administration to ATG16L1[1–249]-expressing cells, suggesting that a minor fraction of the total increase in LC3B-II can be attributed to canonical lipidation (Figure 4H). Altogether, these data show that the increase in lipidated LC3B upon CBK79 administration is primarily mediated by an unconventional route that bypasses the requirement of the ULK1 and PtdIns3K complexes, but that still depends on the ATG12–ATG5-ATG16L1 conjugation complex.

### CBK79 induces proteotoxic stress

**Figure 5.**
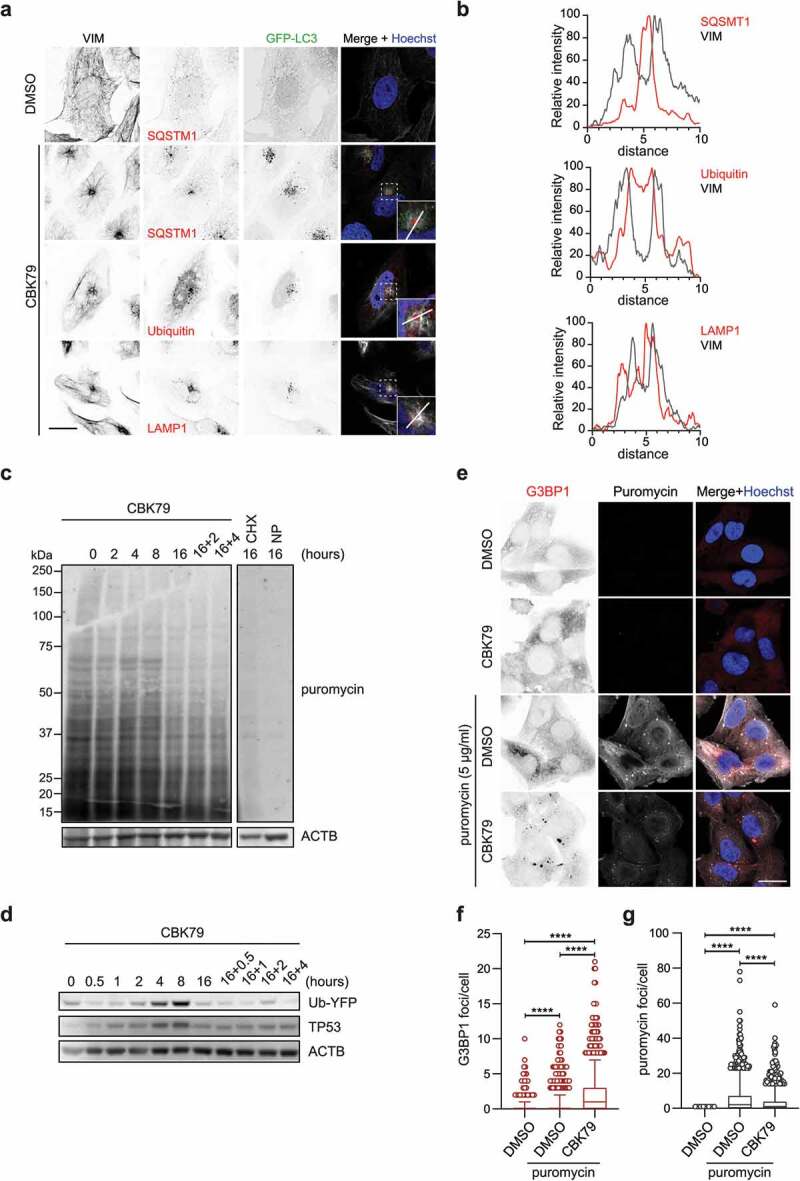
CBK79 induces proteotoxic stress. (A) Representative images of HOS GFP-LC3B cells treated with DMSO 0.1% or CBK79 (10 µM) for 4 h. Cells were fixed and immunostained using antibodies against VIM, SQSTM1, ubiquitin or LAMP1. Scale bar: 20 µm. (B) Line scans at the aggresome sites are shown to visualize the spatial distribution of the indicated proteins (red curves) compared to VIM (gray curves). Intensities are normalized to percentages were 0% = minimum intensity value and 100% = maximum intensity value. (C) MelJuSo Ub-YFP cells were treated with DMSO 0.1% or CBK79 (10 µM) for the indicated timepoints. Two samples were treated for 16 h and then treated with fresh compound solution for the indicated timepoints (“16 h+”). Fifteen minutes before harvesting, puromycin (5 µg/ml) was added to the cells to monitor its incorporation into newly synthesized proteins. Cycloheximide (CHX, 50 µg/ml) was included as control. An untreated sample without puromycin was added (“NP”) as a technical control. Cell lysates were analyzed by immunoblotting with the indicated antibodies. Beta-actin (ACTB) is shown as loading control. Representative blots from one of two independent experiments are shown. (D) MelJuSo Ub-YFP cells were treated with DMSO 0.1% or CBK79 (10 µM) for the indicated timepoints. Two samples were treated for 16 h and then treated with fresh compound solution for the indicated timepoints (“16 h+ re-addition”). Cell lysates were analyzed by immunoblotting with the indicated antibodies. Representative blots from one of three independent experiments are shown. (E) Representative confocal images of MelJuSo Ub-YFP cells treated with DMSO 0.1% or CBK79 (10 µM), alone or in co-treatment with puromycin for 2 h. Cells were fixed and immunostained using antibodies against G3BP1 and puromycin. Scale bar: 20 µm. (F) MelJuSo Ub-YFP cells were pre-treated with DMSO or CBK79 (10 µM) for 30 min and then co-treated with puromycin (5 µg/ml) for 2 h. After nuclei counterstaining with Hoechst 33342, cells were imaged in an automated manner with a widefield fluorescent microscope. The number of G3BP1 foci per cell were quantified using CellProfiler. Pooled data from three independent experiments (DMSO = 1471 cells; DMSO+puromycin = 1521 cells; CBK79+ puromycin = 1467 cells) are shown as box plots with median and 5–95 percentiles. Significant differences are based on adjusted p-values (Kruskal-Wallis [*H* = 1029, df = 2, *p* < 0.0001] with Dunn’s multiple comparisons test). ****p < 0.0001. (G) Cells in (**F**) were analyzed for the number of puromycin foci per cell. Pooled data from three independent experiments (DMSO = 1457 cells; DMSO+puromycin = 1515 cells; CBK79+puromycin = 1472 cells) are shown as box plots with median and 5–95 percentiles. Significant differences are based on adjusted p-values (Kruskal-Wallis [*H* = 1383, df = 2, *p* < 0.0001] with Dunn’s multiple comparisons test). ****p < 0.0001.

Insufficient clearance of misfolded proteins can cause proteotoxic stress, which in turn can lead to the activation of adaptive responses aimed at resolving the critical situation [41]. One such important adaptive response is the sequestration of inherently toxic aggregation-prone proteins in a cytosolic, non-membranous subcellular structure known as the aggresome [42]. Aggresomes are formed by active transport of protein aggregates to the perinuclear region, where they are surrounded by a cage consisting of the intermediate filament VIM (vimentin) [10,11]. Immunostaining of CBK79-treated cells indeed showed the formation of ubiquitin-positive aggresomes that contained the ubiquitin receptor SQSTM1/p62 (sequestosome 1) and were surrounded by VIM, confirming that administration of CBK79 causes loss of proteostasis (Figure 5(A, B); Fig. S5). Moreover, staining of lysosomes using a LAMP1 (lysosomal associated membrane protein 1) antibody showed positioning of lysosomes around the VIM cage (Figure 5(A, B)), an occurrence previously described in aggresomes caused by treatment with proteasome inhibitor [43].

In addition to minimizing the toxic effects of aggregation-prone proteins, proteotoxic stress can also trigger cellular responses to reduce the levels of aberrant proteins by inhibiting the synthesis of new proteins [44]. Puromycin labeling was used to analyze the effect of CBK79 on protein synthesis [45], which revealed that the initial inhibition of the UPS and autophagy was followed by an overall reduction in synthesis of proteins that became prominent 16 h after administration of the compound (Figure 5C). Inhibition of protein synthesis was accompanied by reduced levels of the Ub-YFP reporter substrate and the endogenous substrate TP53/p53, implying that inhibition of protein synthesis successfully reduced the load of UPS substrates (Figure 5D).

Administration of puromycin in combination with compounds that inhibit the UPS has been shown to result in the induction of stress granules, which are small cytosolic granules that contain naked mRNAs and RNA binding proteins [46]. Immunostaining for the RNA binding protein and stress granule marker G3BP1 (G3BP stress granule assembly factor 1) showed that while administration of either puromycin or CBK79 did not induce the formation of stress granules, these structures were formed when cells were exposed to puromycin and CBK79 at the same time (Figure 5(E, F)). Thus, like other compounds that disturb proteostasis, CBK79 induces stress granules in puromycin-sensitized cells. Notably, CBK79 administration strongly reduced the puromycin signal in immunostainings (Figure 5E) and gave rise to fewer puromycin-positive foci than cells that had been incubated with puromycin in the absence of CBK79 (Figure 5G), which is consistent with the decrease in puromycin signal observed by western blotting. We conclude that CBK79 activates cellular stress responses, indicative for induction of proteotoxic stress.

### CBK79 induces the heat shock response

**Figure 6.**
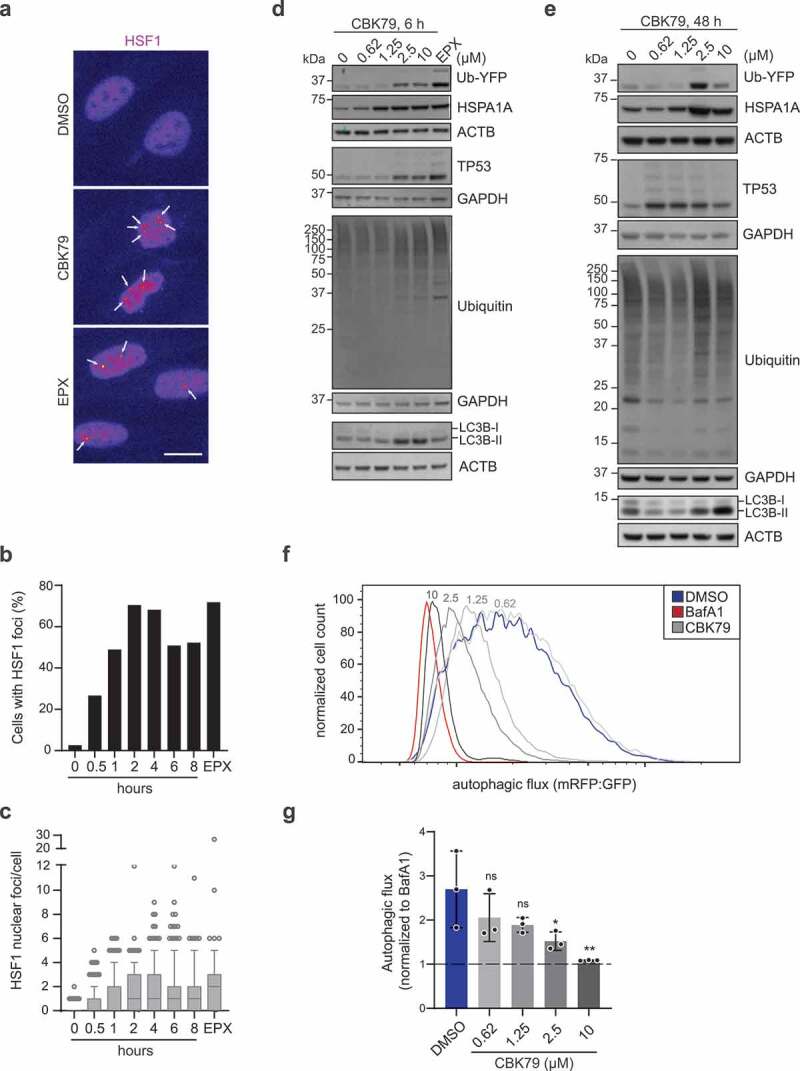
CBK79 induces the heat shock response. (A) HOS GFP-LC3B cells were treated with DMSO 0.1% (8 h), CBK79 10 µM (0 to 8 h; a representative image for the 2 h timepoint is shown or epoxomicin (EPX, 100 nM) for 8 h. Cells were fixed and immunostained with an HSF1 antibody. HSF1 nuclear foci are marked with white arrows. Scale bar: 20 µm. (B) The percentage of cells with HSF1 foci from one of two independent experiments are shown. (C) The number of HSF1 foci per cell were quantified using CellProfiler. Data from one of two independent experiments (n > 200 cells/condition) are shown as box plots with median and 5–95 percentiles. (D) MelJuSo Ub-YFP cells were treated with DMSO 0.1% (0) or the indicated concentrations of CBK79 for 6 h. Cell lysates were analyzed by immunoblotting with the indicated antibodies. Beta-actin (ACTB) is shown as loading control. Representative blots from one of three independent experiments are shown. (E) MelJuSo Ub-YFP cells were treated with DMSO 0.1% (0) or the indicated concentrations of CBK79 for 48 h. Cell lysates were analyzed by immunoblotting with the indicated antibodies. Representative blots from one of three independent experiments are shown. (F) Analysis of autophagic flux in U2OS mRFP-GFP-LC3B cells treated with DMSO 0.1%, bafilomycin A_1_ (BafA1, 100 nM) or CBK79 at the indicated concentrations for 4 h, washed briefly in saponin (0.05%) and analyzed by flow cytometry. Autophagic flux was determined as the ratio of mean mRFP- and mean GFP-fluorescence. A representative histogram from one of three independent experiments is shown. (G) Data from (F) were normalized to BafA1. Data are presented as the mean ± SD of three independent experiments. Significant differences are based on adjusted p-values of multiple comparisons to the DMSO control condition (one-way ANOVA [*F*_4,10_ = 4.860, *p* = 0.0195] with Dunnett’s multiple comparisons test). *p = 0.0388; **p = 0.0064; ns *p* (0.62) = 0.3356; ns *p* (1.25) = 0.1791.

The heat shock response plays an important role in adequate handling of proteotoxic stress as it induces molecular chaperones that prevent protein aggregation, assist in (re-)folding of proteins or redirect terminally misfolded proteins for degradation [47]. HSF1 (heat shock transcription factor 1), which is the central transcriptional regulator of the heat shock response, accumulates in the nucleus when the levels of misfolded proteins exceed a critical threshold [48]. In agreement with induction of a stress response, administration of CBK79 resulted in an increase in nuclear HSF1 (Figure 6A). CBK79 treatment also resulted in accumulation of HSF1 in nuclear stress bodies in a time-dependent fashion (Figure 6(B, C)), arguing that its ability to disturb proteostasis is not confined to the cytosol but also involves the nuclear compartment [49]. Throughout the study, we used relatively high concentrations of CBK79 (5–10 µM) when investigating its effect on the UPS and autophagy whereas we detected effects on cell viability at lower concentrations upon extended exposure. Therefore, we determined the effect of CBK79 on the response to misfolded proteins (by detecting HSPA1A/Hsp70 [heat shock protein family A (Hsp70) member 1A]), the UPS (Ub-YFP and TP53/p53) and autophagy (LC3B-II) in a dose-response experiment. This revealed that induction of HSPA1A/Hsp70 as well as accumulation of Ub-YFP, TP53/p53 and LC3-II can already be detected when MelJuSo cells had been exposed for 6 h to 2.5 µM CBK79 (Figure 6(D, E)). A significant reduction in the autophagic flux was also already evident after a 4 h incubation with 2.5 µM CBK79 (Figure 6(F, G)). We conclude that CBK79 inflicts UPS and autophagy impairment and induces proteotoxic stress already after a short exposure to 2.5 µM CBK79.

### Thermal preconditioning prevents inhibition of the UPS while autophagy impairment persists

**Figure 7.**
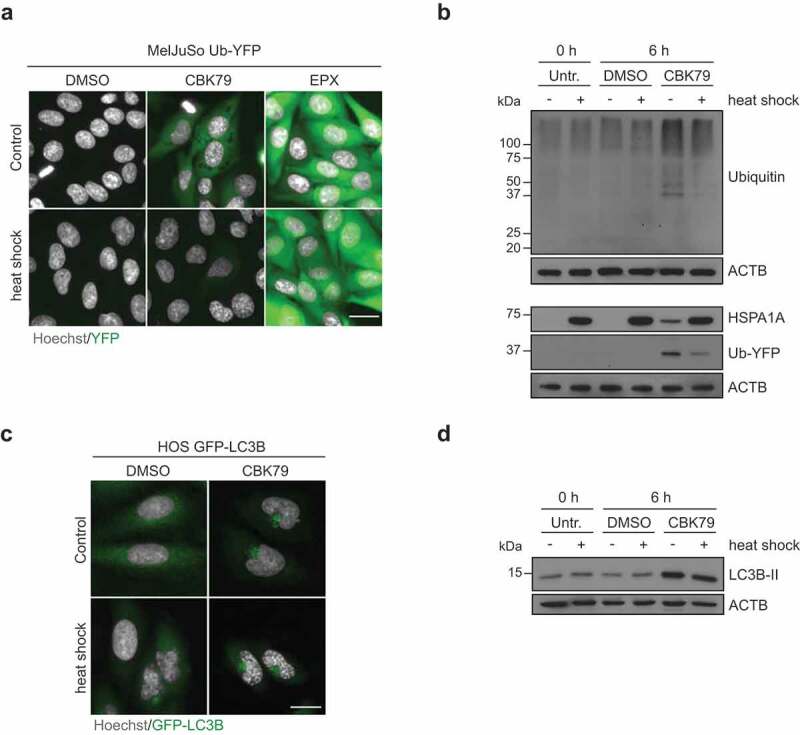
Thermal preconditioning prevents inhibition of the UPS while autophagy impairment persists. (A) Maximum intensity projections of MelJuSo Ub-YFP primed with a 30-min heat shock (43°C), recovered for 8 h, and treated with CBK79 (10 µM), epoxomicin (EPX, 100 nM) or DMSO 0.1% for 6 h. Nuclei were counterstained with Hoechst 33342 and cells imaged in an automated widefield microscope. Scale bar: 20 µm. (B) MelJuSo Ub-YFP were primed with a 30-min heat-shock (43°C), recovered for 8 h, and treated with DMSO 0.1% or CBK79 (10 µM) for 6 h. Cell lysates were analyzed by immunoblotting with the indicated antibodies. Beta-actin (ACTB) is shown as loading control. Representative blots from one of three independent experiments are shown. (C) Maximum intensity projections of HOS GFP-LC3B cells primed with a 30-min heat shock (43°C), recovered for 8 h, and treated with DMSO 0.1% or CBK79 (10 µM) for 4 h. Nuclei were counterstained with Hoechst 33342 and cells imaged in an automated widefield microscope. Scale bar: 20 µm. (D) HOS GFP-LC3B cells were treated as in (**C**). Cell lysates were analyzed by immunoblotting with the indicated antibodies. Representative blots from one of two independent experiments are shown.

Activation of the heat shock response by exposing cells to a brief heat shock renders them resistant to conditions that cause proteotoxic stress, a phenomenon known as thermotolerance [47]. When MelJuSo cells were exposed for 30 min to 43°C prior to administration of CBK79, the accumulation of the Ub-YFP reporter substrate was strongly reduced, while preconditioning had little effect on stabilization of Ub-YFP in cells treated with proteasome inhibitor (Figure 7A). Moreover, the increase in ubiquitin conjugates in response to CBK79 was less pronounced in preconditioned cells, showing that this effect is not limited to the reporter substrates but also applies to endogenous proteasome substrates (Figure 7B). Interestingly, the CBK79-induced appearance of LC3B puncta (Figure 7C) and non-canonical lipidation of LC3B (Figure 7D) were still evident after thermal preconditioning. Thus, while our data suggest that proteotoxic stress contributes to the accumulation of UPS substrates, the effect of CBK79 on autophagy is disconnected from UPS impairment and cannot be counteracted by activation of cellular stress responses.

## Discussion

Although efficient and tightly regulated intracellular degradation is essential for the viability of practically all cells, the increased dependency of malignant cells on these proteolytic systems gives rise to a therapeutic window where curtailing their capacity is lethal for cancer cells without causing substantial harm to other cells [16]. In various studies, the critical roles of the UPS and autophagy in intracellular protein degradation have been explored as therapeutic targets for cancer treatment [18,50]. This has led to the development of clinically approved proteasome inhibitors for the treatment of multiple myeloma and mantle cell lymphoma [51]. On the other hand, chloroquine and its derivative hydroxychloroquine, two FDA-approved drugs that target lysosomal degradation, are being tested in various clinical trials [18]. Our data on the novel compound CBK79 shows that this active molecule has unique features as it inhibits both ubiquitin-dependent and -independent degradation of short-lived proteins by the UPS, as well as the degradation of long-lived proteins by autophagy.

Interference with both proteolytic pathways is an exceptional feature although not unprecedented. A small molecule inhibitor of the ATPase activity of the ubiquitin-selective unfoldase VCP/p97 (valosin containing protein) impaired both the UPS and autophagy resulting in a more rapid induction of apoptosis as compared with proteasome inhibitor [52]. Earlier studies showed that this AAA-ATPase by means of its unfolding activity is involved in ubiquitin-dependent proteasomal degradation of a diverse set of substrates [53], as well as the maturation of autophagosomes [54,55], providing an explanation for the dual activity of this inhibitor. Consistent with its behavior as a ubiquitin-selective complex, inhibition was, however, restricted to ubiquitin-dependent proteasome substrates [52]. This stands in contrast to the activity of CBK79 as this also impairs degradation of the ubiquitin-independent substrate ZsGreen-ODC, suggesting a different mode of action. It is also noteworthy that UPS inhibition by another VCP/p97 inhibitor was accompanied by stimulation of autophagy [56], implying that the ability of VCP/p97 inhibitors to target both pathways may depend on the nature of the inhibitory compound.

While it is clear that the simultaneous impairment of these partially compensatory proteolytic systems will severely challenge the capacity of cells to properly execute protein quality control, the precise mode of action of CBK79 responsible for this dual effect remains to be clarified. A possible cause for the UPS impairment is an acute overload of this proteolytic system with misfolded proteins, which is supported by our observation that thermal preconditioning can prevent CBK79-inflicted UPS impairment. This is in line with several studies that show that various proteotoxic stress conditions can compromise the UPS [24,34,57]. Overwhelming the pool of proteasomes with misfolded proteins, many of which may be hard to degrade due to their propensity to aggregate, is therefore likely to contribute to the observed UPS defect. This may also explain the inhibition of a ubiquitin-independent substrate as these substrates equally depend on sufficient proteasomal capacity for their degradation. As proteasomes, due to the size constraints of the entrance pore, cannot handle protein aggregates, they may be more susceptible to the negative effects of protein aggregates than autophagy [34]. Consistent with this model, thermal preconditioning did not relieve the inhibitory effect of CBK79 on autophagy, suggesting that preventing protein aggregation is of less relevance for autophagy inhibition. This does not exclude the possibility that proteotoxic stress plays a role in CBK79-induced inhibition of autophagy, as critical autophagic proteins may be subject to misfolding rendering them nonfunctional. While the increase in molecular chaperones in thermally preconditioned cells might prevent their aggregation, it may be less successful in restoring their functionality in autophagy.

Interestingly, we found that the formation of LC3B puncta in CBK79-treated cells was primarily facilitated by non-canonical lipidation of LC3B as it was independent of the ULK1 and PtdIns3K complexes that are critical for the formation of autophagosomes. Although surprising, other autophagy inhibitors have also been shown to display broad, poorly characterized unconventional types of LC3 lipidation, suggesting that this is a more common feature among autophagy inhibitors [36,37]. Importantly, the reduced autophagic flux and impaired degradation of long-lived proteins unequivocally shows that this proteolytic pathway is inhibited in CBK79-treated cells.

Except for proteasome inhibitors, attempts to clinically exploit drugs that target proteostasis have been with limited success so far. The novel compound presented in this study shows that it is possible to target both the UPS and autophagy causing a global collapse of intracellular protein quality control. To what extent the accompanying loss of proteostasis caused by CBK79 is a cause or consequence of inhibition of these proteolytic systems remains to be elucidated. However, regardless of the sequence of events that underlies the inhibition of intracellular protein degradation, our study illustrates the potential of disturbing proteostasis by simultaneously interfering with these two central processes in protein quality control.

## Materials and methods

### Cell culture

MelJuSo Ub-YFP cells have been previously described [24]. HOS parental and GFP-LC3B were obtained from Gerald McInerney [58]. U2OS mRFP-GFP-LC3 were obtained from Jeff MacKeigan [59]. HeLa TREx FlpIn WT were a kind gift from A. Tighe and S.S. Taylor (University of Manchester, UK) and used to generate the *ATG13* and *ATG16L1* knockout cell lines, as described below. HEK293 WT cells were obtained from Tamotsu Yoshimori (Osaka University, Japan) and were used to generate *ATG16L1* knockout cell lines reconstituted with either eGFP-ATG16L1β or eGFP-ATG16L1[1–249] as described in [40]. All cells were cultured in DMEM+GlutaMAX (Life Technologies, 31,966–021) supplemented with 10% fetal bovine serum (Life Technologies, 10,270–106) in a humidified incubator at 37°C and 5% CO_2_. All cell lines were routinely tested for *Mycoplasma* infection.

To induce autophagy, cells were treated with 250 nM Torin 1 (Tocris, 4247) or with 500 nM KU-0063794 (Sigma-Aldrich, SML0382). Autophagy was blocked using 10 mM 3-methyladenine (3-MA; Sigma-Aldrich, 189,490), 100 nM bafilomycin A_1_ (BafA1; Enzo Life Sciences, BML-CM110) or 10 µM chloroquine (CQ; Sigma-Aldrich, C6628).

### CRISPR-Cas9 knockout of human ATG16L1 and ATG13

Knockouts of ATG16L1 and ATG13 were generated in HeLa TREx FlpIn cells using sgRNA guides designed using the CRISPR Design Tool through MIT (crispr.mit.edu). The following guides were selected: *ATG16L1* guide 4 (5`-TTA CGT GGC TGC TCT GCT GA), *ATG16L1* guide 5 (5`-GCC ACA TCT CGG AGC AAC TG) and *ATG13* guide 1 (5`- TTT ACC CAA TCT GAA CCC GT). The sgRNA guides were synthesized (Sigma-Aldrich) and cloned into the pSpCas9 (BB)-2A-Puro (pX459) V2.0 plasmid (Addgene, 62,988; deposited by Feng Zhang). Cells were transfected using XtremeGene9 (Roche, obtained via Sigma-Aldrich, 6,365,787,001) according to the manufacturer’s protocol. After 24 h, cells were treated with selection medium containing 2.0 μg/ml puromycin and fresh selection medium was provided every 2–3 days. After 7 days puromycin resistant cells were seeded as single cell/well in a 96-well plate through serial dilution. Individual clones were isolated and expanded before knockouts were confirmed via western blotting.

### Chemical screen

For the primary screen, MelJuSo Ub-YFP cells were seeded in black with clear bottom 384-well plates (Falcon, 35–3962) by distributing 30 µl/well using Matrix WellMate™ (Thermo Fisher Scientific; Waltham, MA, USA) and incubated overnight at 37°C in a 5% CO_2_ atmosphere. The following day, cells were exposed to compounds at a final concentration of 10 µM. The compound stocks (in DMSO) were first diluted in DMEM at a concentration of 20 µM, to then distribute 30 µl/well to achieve the desired final concentration using the Biomek 384-well NX robot (Beckman Coulter). Epoxomicin (Enzo Life Sciences, BML-PI127) was added to specific wells (8 wells per plate) as a positive control at a final concentration of 100 nM. DMSO controls (24 wells per plate) at 0.1% concentration were used as negative controls. The cells were incubated with the compounds for 19–20.5 h before Hoechst 33342 (Life Technologies, H3570) was added at a final concentration of 2 µg/ml. Plates were imaged using the high-content screen microscope ArrayScan VTi (Thermo Fisher Scientific) coupled to Cellomics. Images were automatically taken using 10x magnification and two randomized images/well were taken. Using Cellomics, an algorithm was created to first segment the nuclei based on the Hoechst staining, and then measure the mean YFP intensity per cell including the Hoechst area + 2 pixels. The final measurements obtained were total cell count/well and mean YFP intensity/well calculated by the sum of all the YFP-intensities per cell divided by the total number of nuclei. The z’-factor was used as a measurement of quality control. For each plate of the screen, the z’-factor was calculated based on the YFP readout using the following formula:

$$z{\;}^{\prime }=1-\frac{\left(3\sigma_{posctrl}+3\sigma_{negctrl}\right)}{\left(\mu_{posctrl}-\mu_{negctrl}\right)}$$

where $\sigma_{posctrl}$ is the standard deviation of the positive control (epoxomicin), $\sigma_{negctrl}$ is the standard deviation of the negative control (DMSO) and $\mu_{posctrl}-\mu_{negctrl}$ is the difference between the mean value for the positive and negative controls. The average z’-factor of the screen was 0.5387.

### Compound library

The compound library was provided by the Laboratories for Chemical Biology Umeå (LCBU), a part of the Chemical Biology Consortium Sweden (CBCS). This chemical collection, named LCBU primary screening set, was constructed by selecting and purchasing compounds from ChemBridge representing a diverse selection biased toward lead-like and drug-like properties with regards to molecular weight, hydrogen bond donors/acceptors and LogP. The compounds were stored at 1 or 5 mM concentration in DMSO in desiccators at room temperature (RT).

### Filtering of hit compounds

In order to identify and remove potentially problematic compounds, the hits from the primary screen were assessed for Lipinski’s rule of 5 violations and passed through filters built in the analytics platform KNIME (KNIME AG) by Evert Homan (Karolinska Institutet) to detect unwanted substructures and known PAINS (https://hub.knime.com/evert.homan_scilifelab.se/spaces/Public/latest/~MrK3gq6WBD5fAfgy/).

### Long‐lived protein degradation assay

Cells were added at a density of 8.8 × 10^4^ cells/ml in a final volume of 400 μl. Cells were labeled by the addition of 0.125 μCi/ml L‐[^14^C] valine (Perkin Elmer, NEC291EU050UC) to the medium for 24 h, followed by two washes and 16 h chase in medium containing 10 mM nonradioactive l‐valine (Sigma-Aldrich, V0513), to allow degradation of short‐lived proteins. Subsequently, cells were washed and subjected to the indicated treatments in medium containing 10 mM nonradioactive l‐valine for 4 h. Radioactivity in the acid‐soluble and acid‐insoluble fractions was measured using Ultima Gold LSC cocktail (Perkin Elmer, obtained via Sigma-Aldrich, L8286) and the liquid scintillation counter Tri‐Carb 3100 T (Perkin Elmer; Waltham, MA, USA).

### GFP-LC3B puncta assay

For analysis of living cells, HOS GFP-LC3B cells were seeded and treated in a 96-well imaging plate (BD Falcon™, 35–3219). Hoechst 33342 (12 μg/mL in 1 x PBS [Statens Veterinärmedicinska Anstalt, 992442]) was added to the cells at a final concentration of 2 μg/mL and incubated for 30 min. Prior to imaging, medium was replaced by Leibovitz’s L-15 medium (Life Technologies, 11415–064) and four to six sites per well imaged without delay using an ImageXpress automated widefield microscope (Molecular Devices) equipped with a 20x objective. Analysis of immunolabeled cells was done similarly. The number of cytoplasmic GFP-LC3B puncta per cell was quantified using CellProfiler Software 2.1.1. (Broad Institute).

### Ub-YFP levels by microscopy

For the analysis of the Ub-YFP reporter in living cells, MelJuSo cells stably expressing the Ub-YFP reporter were seeded and treated in a 96-well plate. Hoechst 33342 (12 μg/mL in 1 x PBS) was added to the cells at a final concentration of 2 μg/mL and incubated for 30 min. Four to nine sites per well were imaged using an ImageXpress automated widefield microscope (Molecular Devices) equipped with a 20x objective. The YFP intensity per cell and the percentage of YFP-positive cells were quantified using the MetaXpress software. The in-built pipeline Multiwavelength Scoring analysis was performed. Briefly, nuclei segmentation was based on the Hoechst staining, with minimum intensity and nuclei size thresholds defined by the user tailored to each experiment. The mean YFP intensity per cell reported throughout the manuscript represents the mean YFP intensity per cell in the nucleus. Based on a tailored threshold defined by the user, the percentage of YFP positive cells was also obtained.

### Flow cytometry analysis of autophagic flux

For analysis of autophagic flux by flow cytometry, U2OS mRFP-GFP-LC3B cells were seeded and treated in 6-well plates. Cells were harvested with trypsin and washed with PBS. Extraction of non-autophagosome associated mRFP-GFP-LC3B was done by briefly washing with 0.05% saponin (Sigma-Aldrich, 47036) in PBS [58]. Using the BD FACSAria III (BD Biosciences; Franklin Lakes, NJ, USA), 30,000 events were captured per experiment and cellular mRFP- and GFP-intensities measured using the 488-nm and 561-nm lasers. Data were analyzed using FlowJo (Treestar Inc.). Autophagic flux was determined as the ratio between mean intensities of mRFP and GFP.

### Immunofluorescent labeling

Cells were seeded on 18-mm coverslips or in 96-well imaging plates and treated as indicated. Cells were fixed in 4% paraformaldehyde in 1 x PBS for 20 min; permeabilized using 0.2% Triton X-100 (Sigma-Aldrich, T8787) or 0.2% saponin in 1 x PBS for 15 min (in the case of endogenous LC3B staining) and incubated with 100 mM glycine in 1 x PBS for 10 min. Unspecific binding was blocked using 3% BSA (Sigma-Aldrich, A7030) in 1 x PBS for 30 min. All steps were performed at RT. The following primary antibodies were diluted in BSA 3% in PBS and incubated overnight at 4°C: anti-LC3B (MBL Life Science, PM036), anti-LAMP1 (Santa Cruz Biotechnology, sc-20011), anti-VIM/vimentin (Sigma-Aldrich, AB5733), anti-SQSTM1/p62 (Santa Cruz Biotechnology, sc-25575), anti-G3BP1 (Invitrogen, PA5–29455), anti-puromycin (Sigma-Aldrich, MABE343), anti-ubiquitin (FK2, conjugated ubiquitin; Enzo Life Sciences, PW8810), anti-HSF1 (Cell Signaling Technologies, 4356). Goat anti-mouse or goat/donkey anti-rabbit IgG coupled to Alexa Fluor 488, 546 or 647 (Life Technologies, A-11029, A-11030, A-21235, A-11035, A-27040) were diluted 1:500 in BSA 3% in PBS. Nuclear staining was performed using Hoechst 33342 1:5000 in PBS for 10 min. After washing with PBS, the plate was imaged using the ImageXpress microscope (Molecular Devices; San Jose, CA, USA) with a 20x objective or mounted and imaged in the LSM880 confocal microscope and Zeiss ZEN software (Zeiss; Oberkochen, Germany) with a 63x objective.

The number of puromycin foci in the cytoplasm per cell, the number of G3BP1 foci in the cytoplasm per cell and the HSF1 foci in the nucleus per cell were quantified using CellProfiler Software 2.1.1. (Broad Institute). ImageJ (https://imagej.nih.gov/ij/) was used for measuring intensities in line scans and for the processing of representative images.

### Cell viability assay

Cells were seeded into clear-bottom, white 96-well plates at 4500 (MelJuSo) cells per well. Sixteen h after seeding (ca 60% confluency), cells were treated in technical triplicates with the indicated compound concentrations in a 9-point serial dilution (unless otherwise indicated in the figure legends, starting from 10 µM, 2-fold serial dilutions were performed). DMSO at 0.1% was used as control. After 24, 48 or 72 h of incubation, an ATP-based cell viability assessment was performed using CellTiter-Glo (Promega, G7573) following the manufacturer’s instructions. Briefly, the plate was taken out of the incubator approximately 30 min before adding the CellTiter-Glo reagent. To aid cell lysis, the plate was placed in the plate reader FLUOStar OMEGA (BMG Labtech; Ortenberg, Germany) and orbital shaking at 500 rpm was performed for 2 min. Afterward, the plate was incubated 10 min in the dark before the luminescent signals were detected. Three wells with medium + CellTiter-Glo without cells were used as blanks, and the average luminescent signal of these wells was subtracted from all the values.

### Western blotting

Equal amounts of cells were lysed in 1X SDS sample buffer (0.3 M Tris-HCl, pH 6.8, 2% SDS, 17.5% glycerol [Sigma-Aldrich, G5516], bromophenol blue [Sigma-Aldrich, B0126]) containing 10% NuPAGE reducing agent (Thermo Fisher Scientific, NP0004) and lysates were boiled at 95°C for 5 min. Cell protein extracts were resolved by Bis-Tris polyacrylamide gel electrophoresis gels (Thermo Fisher Scientific, 4–12% gradient gels [NP0323] or 12% gels [NP0343] for LC3B blots) and run in either 1X MOPS (Thermo Fisher Scientific, NP0001) or for LC3B blots in 1X MES buffer (Thermo Fisher Scientific, NP0002). Proteins were transferred onto PVDF 0.45 µm or nitrocellulose membranes (GE Healthcare, 10600023) in a Tris-glycine transfer buffer (25 mM Tris, 192 mM glycine) containing 20% methanol. After blocking in Tris-buffered saline (TBS; Statens Veterinärmedicinska Anstalt 303252), 5% nonfat milk containing 0.1% Tween-20 (Sigma-Aldrich, P9416), membranes were incubated with primary antibodies, washed with TBS-0.1% Tween-20 and incubated with secondary HRP-linked antibodies (GE Healthcare, NA934V and NA931V). Detection was performed by enhanced chemiluminiscence (Amersham ECL reagents; GE Healthcare, RPN2106) on Medical X-ray films (Super XR, Fujifilm). Alternatively, secondary antibodies coupled to near-infrared fluorescent dyes (LI-COR, 926–68070 and 926–68071) were used, and membranes scanned with an Odyssey scanner (LI-COR, Lincoln, NE, USA) and analyzed with Image Studio Lite analysis software version 5.2 (LI-COR).

The following antibodies were used: anti-GFP (Abcam, ab290), anti-HSPA1A/Hsp70 (C92F3A-5; Enzo Life Sciences, ADI-SPA-810), anti-GAPDH (Abcam, ab9485), anti-ACTB/β-actin (Abcam, ab8226), anti-TP53/p53 (DO-1; Santa Cruz Biotechnology, sc-126), anti-HIF1A/HIF1α (GeneTex, GTX127309), anti-LC3B (Sigma-Aldrich, L7543), anti-ATG16L1 (MBL Life Science, PM040).

### Proteasome activity assay

After treatments, cells were trypsinized and lysed (25 mM HEPES pH 7.2 [Thermo Fisher Scientific, 15630–056], 50 mM NaCl, 1 mM MgCl_2_ [Sigma-Aldrich, M8266], 1 mM ATP [Sigma-Aldrich, A1852], 1 mM DTT, 10% glycerol, 1% Triton X-100) for 30 min at 4°C. Ten μg protein in 100 μl reaction buffer were mixed with 100 μM Suc-Leu-Leu-Val-Tyr-AMC (Affiniti, P802). In one well, 500 nM epoxomicin (Enzo Life Sciences, BML-PI127) was added additionally. Samples were analyzed in a microplate reader FLUOStar OPTIMA (BMG Labtech; Ortenberg, Germany) at 380 nm/440 nm every min for 1 h.

### Statistical analysis

Statistical analyses were performed using GraphPad Prism version 8.3. To test for Gaussian distribution, the D’Agostino & Pearson or Shapiro-Wilk normality test (for smaller sample sizes) were used. If the normality test was passed, data were analyzed by Student’s unpaired two-tailed t-test (two groups) or by ANOVA (more than two groups). If the data were not normally distributed, statistical analysis was performed using the nonparametric Mann-Whitney test (two groups) or Kruskal-Wallis test for multiple comparisons, with Dunnett’s or Tukey’s test to adjust for multiple comparisons. Data are shown as mean ± SD (standard deviation), unless stated otherwise, as indicated in each figure legend. The following p-values were considered significant: *p ≤ 0.05; **p ≤ 0.01; ***p ≤ 0.001; ****p ≤ 0.0001.

### EC_50_ or IC_50_ values

Dose-response curves were fitted in GraphPad Prism by a non-linear regression analysis using a four-parameter logistic curve fit to estimate IC_50_ (cell viability) or EC_50_ (Ub-YFP accumulation) values. In the case of the IC_50_, bottom values were constrained to 0.

### General synthetic procedures for compounds

All reactions were carried out using dry solvents and anhydrous condition, unless otherwise stated. All of solvents were dried with PPT’s solvent purification systems. Liquid chromatography/mass spectrometry (LC/MS) was carried out with either an Agilent system (Santa Clara, CA, USA) or a Waters system (Milford, MA, USA), as explained below.

Agilent 1260 binary LC system equipped with an Agilent EC-C18 column (3.0 x 50 mm, 2.7 µm), eluted with a linear gradient of CH_3_CN in H_2_O, both of which contained trifluoroacetic acid (0.1%). A flow rate of 0.7 mL/min was used and detection was performed at 214 and 254 nm. Low resolution mass spectra (LRMS) data were obtained on an Agilent 6130 Quadrupole LC/MS using positive and negative electrospray ionization.

Waters LC system equipped with an Xterra C-18 column (50 x 19 mm, 5 µm, 125 Å), eluted with a linear gradient of CH_3_CN in H_2_O, both of which contained formic acid (0.2%). A flow rate of 1.5 mL/min was used and detection was performed at 214 and 254 nm, and with positive and negative electrospray mass analysis. Low resolution mass spectra (LRMS) data was obtained on a Water micromass ZQ 2000 using positive and negative electrospray ionization.

Flash column chromatography was performed on silica gel (60 Å, 230–400 mesh). Preparative HPLC was performed using VP 250/21 NUCLEODUR C-18, HTEC, 5 µm column on a GILSON 333/334 Prep-Scale system with a flow rate of 20 mL/min, detection at 254 nm, and CH_3_CN/H_2_O eluent system. ^1^H and ^13^C NMR spectra were recorded at 298° K with a Brucker DRX-400 spectrometer at 400 MHz and 100 MHz, or Brucker DRX-600 spectrometer at 600 MHz and 150 MHz, respectively, and calibrated using the residual peak of the solvent as internal standard CDCl_3_ (CHCl_3_ δ_H_ 7.26 ppm, CDCl_3_ δ_C_ 77.16 ppm) or DMSO_d6_ (DMSO_d5_ δ_H_ 2.50 ppm, DMSO_d6_ δ_C_ 39.52 ppm).

Specific information for each compound is provided in the supplementary material.

## Supplementary Material

Supplemental Material

## References

[1] Sala AJ, Bott LC, Morimoto RI. Shaping proteostasis at the cellular, tissue, and organismal level. J Cell Biol. 2017 May 1;216(5):1231–1241. 28400444 10.1083/jcb.201612111 PMC5412572

[2] Schrader EK, Harstad KG, Matouschek A. Targeting proteins for degradation. Nat Chem Biol. 2009 Nov;5(11):815–822. 19841631 10.1038/nchembio.250 PMC4228941

[3] Verhoef LG, Lindsten K, Masucci MG, et al. Aggregate formation inhibits proteasomal degradation of polyglutamine proteins. Hum Mol Genet. 2002;11(22):2689–2700. 12374759 10.1093/hmg/11.22.2689

[4] Sherman MY, Goldberg AL. Cellular defenses against unfolded proteins: a cell biologist thinks about neurodegenerative diseases. Neuron. 2001;29(1):15–32. 11182078 10.1016/s0896-6273(01)00177-5

[5] Dikic I, Elazar Z. Mechanism and medical implications of mammalian autophagy. Nat Rev Mole Cell Biol. 2018 Jun;19(6):349–364. 10.1038/s41580-018-0003-4 29618831

[6] Schaaf MB, Keulers TG, Vooijs MA, et al. LC3/GABARAP family proteins: autophagy-(un)related functions. FASEB J. 2016 Dec;30(12):3961–3978. 27601442 10.1096/fj.201600698R

[7] Lum JJ, DeBerardinis RJ, Thompson CB. Autophagy in metazoans: cell survival in the land of plenty. Nat Rev Mole Cell Biol. 2005 6;Jun(6):439–448. 10.1038/nrm1660 15928708

[8] Hara T, Nakamura K, Matsui M, et al. Suppression of basal autophagy in neural cells causes neurodegenerative disease in mice. Nature. 2006 Apr 19;441(7095):885–889. 16625204 10.1038/nature04724

[9] Komatsu M, Waguri S, Chiba T, et al. Loss of autophagy in the central nervous system causes neurodegeneration in mice. Nature. 2006 Jun 15;441(7095):880–884. 16625205 10.1038/nature04723

[10] Garcia-Mata R, Bebok Z, Sorscher EJ, et al. Characterization and dynamics of aggresome formation by a cytosolic GFP-chimera. J Cell Biol. 1999 Sept 20;146(6):1239–1254. 10491388 10.1083/jcb.146.6.1239 PMC2156127

[11] Johnston JA, Ward CL, Kopito RR. Aggresomes: a cellular response to misfolded proteins. J Cell Biol. 1998 Dec 28;143(7):1883–1898. 9864362 10.1083/jcb.143.7.1883 PMC2175217

[12] Bucciantini M, Giannoni E, Chiti F, et al. Inherent toxicity of aggregates implies a common mechanism for protein misfolding diseases. Nature. 2002;416(6880):507–511. 11932737 10.1038/416507a

[13] Guang MHZ, Kavanagh EL, Dunne LP, et al. Targeting proteotoxic stress in cancer: a review of the role that protein quality control pathways play in oncogenesis. Cancers (Basel). 2019 Jan 9;11(1):66. 10.3390/cancers11010066 PMC6356294 30634515

[14] Adams J. Proteasome inhibitors as new anticancer drugs. Curr Opin Oncol. 2002;14(6):628–634. 12409653 10.1097/00001622-200211000-00007

[15] Galluzzi L, Pietrocola F, Bravo-San Pedro JM, et al. Autophagy in malignant transformation and cancer progression. EMBO J. 2015 Apr 1;34(7):856–880. 25712477 10.15252/embj.201490784 PMC4388596

[16] Brancolini C, Iuliano L. Proteotoxic stress and cell death in cancer cells. Cancers (Basel). 2020 Aug 23;12(9):2385. 10.3390/cancers12092385 PMC7563887 32842524

[17] Fricker LD. Proteasome inhibitor drugs. Annu Rev Pharmacol Toxicol. 2020 Jan 6;60:457–476. 31479618 10.1146/annurev-pharmtox-010919-023603

[18] Levy JMM, Towers CG, Thorburn A. Targeting autophagy in cancer. Nat Rev Cancer. 2017 Sep;17(9):528–542. 28751651 10.1038/nrc.2017.53 PMC5975367

[19] Lamark T, Johansen T. Autophagy: links with the proteasome. Curr Opin Cell Biol. 2010 Apr;22(2):192–198. 19962293 10.1016/j.ceb.2009.11.002

[20] Wang XJ, Yu J, Wong SH, et al. A novel crosstalk between two major protein degradation systems: regulation of proteasomal activity by autophagy. Autophagy. 2013 Oct;9(10):1500–1508. 23934082 10.4161/auto.25573

[21] Albornoz N, Bustamante H, Soza A, et al. Cellular responses to proteasome inhibition: molecular mechanisms and beyond. Int J Mol Sci. 2019 Jul 10;20(14):3379. 10.3390/ijms20143379 PMC6678303 31295808

[22] Pandey UB, Nie Z, Batlevi Y, et al. HDAC6 rescues neurodegeneration and provides an essential link between autophagy and the UPS. Nature. 2007 Jun 14;447(7146):859–863. 17568747 10.1038/nature05853

[23] Liu D, Gao M, Yang Y, et al. Inhibition of autophagy promotes cell apoptosis induced by the proteasome inhibitor MG-132 in human esophageal squamous cell carcinoma EC9706 cells. Oncol Lett. 2015 May;9(5):2278–2282. 26137056 10.3892/ol.2015.3047 PMC4467331

[24] Menendez-Benito V, Verhoef LG, Masucci MG, et al. Endoplasmic reticulum stress compromises the ubiquitin-proteasome system. Hum Mol Genet. 2005;14(19):2787–2799. Epub 2005 Aug 15. 16103128 10.1093/hmg/ddi312

[25] Dantuma NP, Lindsten K, Glas R, et al. Short-lived green fluorescent proteins for quantifying ubiquitin/proteasome-dependent proteolysis in living cells. Nat Biotechnol. 2000;18(5):538–543. 10802622 10.1038/75406

[26] Johnson ES, Ma PC, Ota IM, et al. A proteolytic pathway that recognizes ubiquitin as a degradation signal. J Biol Chem. 1995;270(29):17442–17456. 7615550 10.1074/jbc.270.29.17442

[27] Gierisch ME, Giovannucci TA, Dantuma NP. Reporter-based screens for the ubiquitin/proteasome system. Front Chem. 2020;8:64. 32117887 10.3389/fchem.2020.00064 PMC7026131

[28] Lipinski CA, Lombardo F, Dominy BW, et al. Experimental and computational approaches to estimate solubility and permeability in drug discovery and development settings. Adv Drug Deliv Rev. 2001 Mar 1;46(1–3):3–26. 11259830 10.1016/s0169-409x(00)00129-0

[29] Baell JB, Holloway GA. New substructure filters for removal of pan assay interference compounds (PAINS) from screening libraries and for their exclusion in bioassays. J Med Chem. 2010 Apr 8;53(7):2719–2740. 20131845 10.1021/jm901137j

[30] Walters WP, Namchuk M. Designing screens: how to make your hits a hit. Nat Rev Drug Discov. 2003 Apr;2(4):259–266. 12669025 10.1038/nrd1063

[31] Fang S, Jensen JP, Ludwig RL, et al. Mdm2 is a RING finger-dependent ubiquitin protein ligase for itself and p53. J Biol Chem. 2000 Mar 24;275(12):8945–8951. 10722742 10.1074/jbc.275.12.8945

[32] Huang LE, Gu J, Schau M, et al. Regulation of hypoxia-inducible factor 1alpha is mediated by an O_2_-dependent degradation domain via the ubiquitin-proteasome pathway. Proc Natl Acad Sci U S A. 1998 Jul 7;95(14):7987–7992. 9653127 10.1073/pnas.95.14.7987 PMC20916

[33] Murakami Y, Matsufuji S, Kameji T, et al. Ornithine decarboxylase is degraded by the 26S proteasome without ubiquitination. Nature. 1992 Dec 10;360(6404):597–599. 1334232 10.1038/360597a0

[34] Bence NF, Sampat RM, Kopito RR. Impairment of the ubiquitin-proteasome system by protein aggregation. Science. 2001;292(5521):1552–1555. 11375494 10.1126/science.292.5521.1552

[35] Klionsky DJ, Abdel-Aziz AK, Abdelfatah S, et al. Guidelines for the use and interpretation of assays for monitoring autophagy (4^th^ edition). Autophagy. 2021 Jan;17(1):1–382. 33634751 10.1080/15548627.2020.1797280 PMC7996087

[36] Jacquin E, Leclerc-Mercier S, Judon C, et al. Pharmacological modulators of autophagy activate a parallel noncanonical pathway driving unconventional LC3 lipidation. Autophagy. 2017 May 4;13(5):854–867. 28296541 10.1080/15548627.2017.1287653 PMC5446083

[37] Liu Y, Luo X, Shan H, et al. Niclosamide Triggers Non-Canonical LC3 Lipidation. Cells. 2019 Mar 15;8(3):248. 10.3390/cells8030248 PMC6468753 30875964

[38] Florey O, Overholtzer M. Autophagy proteins in macroendocytic engulfment. Trends Cell Biol. 2012 Jul;22(7):374–380. 22608991 10.1016/j.tcb.2012.04.005 PMC3383932

[39] Klionsky DJ, Emr SD. Autophagy as a regulated pathway of cellular degradation. Science. 2000 Dec 1;290(5497):1717–1721. 11099404 10.1126/science.290.5497.1717 PMC2732363

[40] Lystad AH, Carlsson SR, de la Ballina LR, et al. Distinct functions of ATG16L1 isoforms in membrane binding and LC3B lipidation in autophagy-related processes. Nat Cell Biol. 2019 Mar;21(3):372–383. 30778222 10.1038/s41556-019-0274-9 PMC7032593

[41] Santiago AM, Goncalves DL, Morano KA. Mechanisms of sensing and response to proteotoxic stress. Exp Cell Res. 2020 Oct 15;395(2):112240. 32827554 10.1016/j.yexcr.2020.112240 PMC7541750

[42] Kopito RR. Aggresomes, inclusion bodies and protein aggregation. Trends Cell Biol. 2000 Dec;10(12):524–530. 11121744 10.1016/s0962-8924(00)01852-3

[43] Zaarur N, Meriin AB, Bejarano E, et al. Proteasome failure promotes positioning of lysosomes around the aggresome via local block of microtubule-dependent transport. Mol Cell Biol. 2014 Apr;34(7):1336–1348. 24469403 10.1128/MCB.00103-14 PMC3993571

[44] Pakos-Zebrucka K, Koryga I, Mnich K, et al. The integrated stress response. EMBO Rep. 2016 Oct;17(10):1374–1395. 27629041 10.15252/embr.201642195 PMC5048378

[45] Schmidt EK, Clavarino G, Ceppi M, et al. SUnSET, a nonradioactive method to monitor protein synthesis. Nat Methods. 2009 Apr;6(4):275–277. 19305406 10.1038/nmeth.1314

[46] Bounedjah O, Desforges B, Wu TD, et al. Free mRNA in excess upon polysome dissociation is a scaffold for protein multimerization to form stress granules. Nucleic Acids Res. 2014 Jul;42(13):8678–8691. 25013173 10.1093/nar/gku582 PMC4117795

[47] Mogk A, Bukau B, Kampinga HH. Cellular handling of protein aggregates by disaggregation machines. Mol Cell. 2018 Jan 18;69(2):214–226. 29351843 10.1016/j.molcel.2018.01.004

[48] Pincus D. Regulation of Hsf1 and the heat shock response. Adv Exp Med Biol. 2020;1243:41–50. 32297210 10.1007/978-3-030-40204-4_3

[49] Biamonti G, Vourc’h C. Nuclear stress bodies. Cold Spring Harb Perspect Biol. 2010 Jun;2(6):a000695. 20516127 10.1101/cshperspect.a000695 PMC2869524

[50] Park J, Cho J, Song EJ. Ubiquitin-proteasome system (UPS) as a target for anticancer treatment. Arch Pharm Res. 2020 Nov;43(11):1144–1161. 33165832 10.1007/s12272-020-01281-8 PMC7651821

[51] Kisselev AF, Van Der Linden WA, Overkleeft HS. Proteasome inhibitors: an expanding army attacking a unique target. Chem Biol. 2012 Jan 27;19(1):99–115. 22284358 10.1016/j.chembiol.2012.01.003 PMC3503453

[52] Chou TF, Brown SJ, Minond D, et al. Reversible inhibitor of p97, DBeQ, impairs both ubiquitin-dependent and autophagic protein clearance pathways. Proc Natl Acad Sci U S A. 2011 Mar 22;108(12):4834–4839. 21383145 10.1073/pnas.1015312108 PMC3064330

[53] Bodnar NO, Rapoport TA. Molecular mechanism of substrate processing by the Cdc48 ATPase complex. Cell. 2017 May 4;169(4):722–735 e9. 28475898 10.1016/j.cell.2017.04.020 PMC5751438

[54] Ju JS, Fuentealba RA, Miller SE, et al. Valosin-containing protein (VCP) is required for autophagy and is disrupted in VCP disease. J Cell Biol. 2009 Dec 14;187(6):875–888. 20008565 10.1083/jcb.200908115 PMC2806317

[55] Beskow A, Grimberg KB, Bott LC, et al. A conserved unfoldase activity for the p97 AAA-ATPase in proteasomal degradation. J Mol Biol. 2009 Dec 11;394(4):732–746. 19782090 10.1016/j.jmb.2009.09.050

[56] Anderson DJ, Le Moigne R, Djakovic S, et al. Targeting the AAA ATPase p97 as an approach to treat cancer through disruption of protein homeostasis. Cancer Cell. 2015 Nov 9;28(5):653–665. 26555175 10.1016/j.ccell.2015.10.002 PMC4941640

[57] Salomons FA, Menendez-Benito V, Bottcher C, et al. Selective accumulation of aggregation-prone proteasome substrates in response to proteotoxic stress. Mol Cell Biol. 2009 Apr;29(7):1774–1785. 19158272 10.1128/MCB.01485-08 PMC2655608

[58] Eng KE, Panas MD, Karlsson Hedestam GB, et al. A novel quantitative flow cytometry-based assay for autophagy. Autophagy. 2010 Jul;6(5):634–641. 20458170 10.4161/auto.6.5.12112

[59] Holland P, Torgersen ML, Sandvig K, et al. LYST affects lysosome size and quantity, but not trafficking or degradation through autophagy or endocytosis. Traffic. 2014 Dec;15(12):1390–1405. 25216107 10.1111/tra.12227

