# Supplementary material for: Identification of a novel compound that simultaneously impairs the ubiquitin-proteasome system and autophagy
Source: Autophagy. 2021 Nov 5;18(7):1486–502. doi: 10.1080/15548627.2021.1988359 (PMC9298443; doi:10.1080/15548627.2021.1988359)
Supplement: Supplemental Material [file KAUP_A_1988359_SM3127.pdf]

Supplementary Material

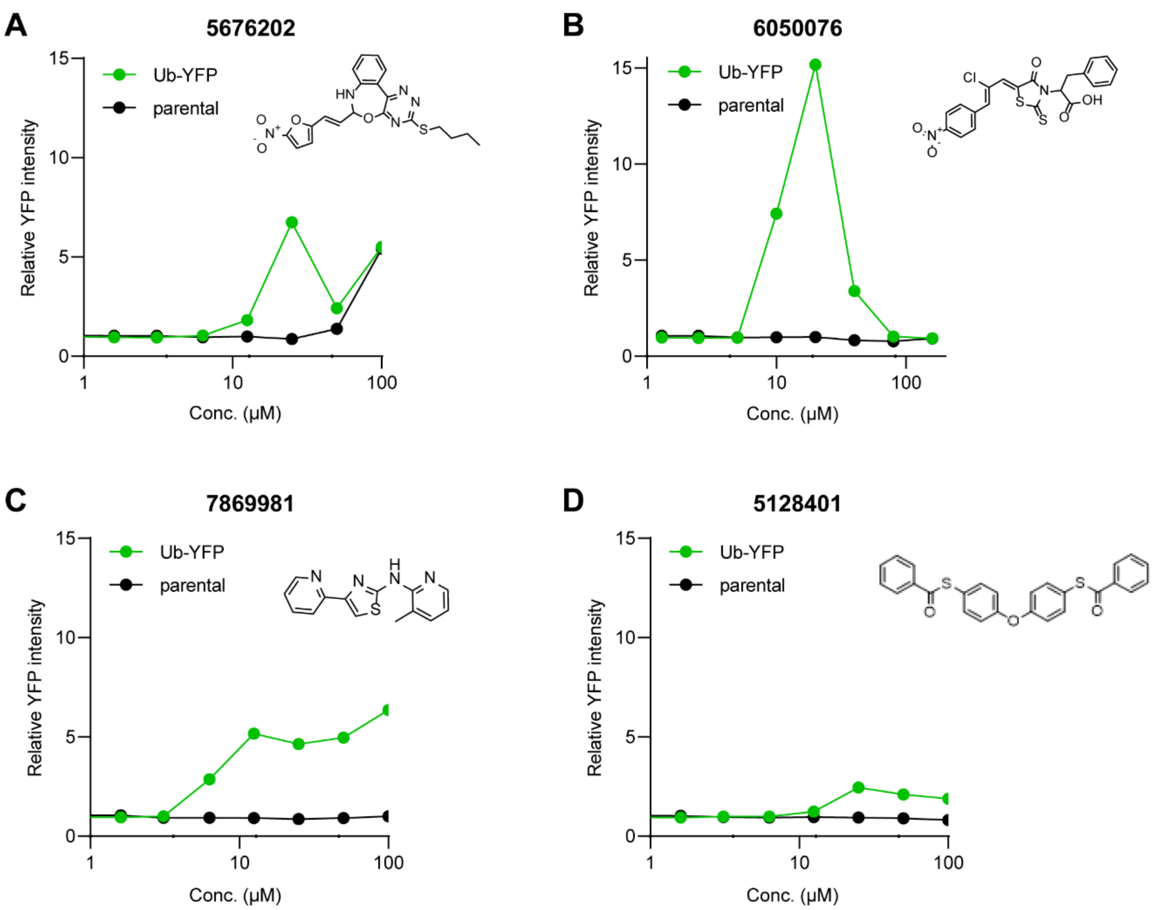

Figure S1. Chemical structures and dose-response curves of the final 4 hits of the primary screen. Compound IDs are shown as identified in the ChemBridge library. Compounds were tested in a dose-response experiment, ranging from 100 μM down to 0.8 μM (except for compound 6050076, tested from 160 μM to 1.3 μM) for 20 h. DMSO concentrations matching the highest compound concentration tested were used as controls. The compounds were tested in the Ub-YFP expressing cell line as well as the parental MelJuSo cell line to exclude autofluorescent compounds. Nuclei were stained with Hoechst 33342 and cells were directly imaged live using the automated widefield microscope ArrayScan VTi coupled to the Cellomics software. Data are represented as the mean YFP intensity per cell relative to the DMSO control.

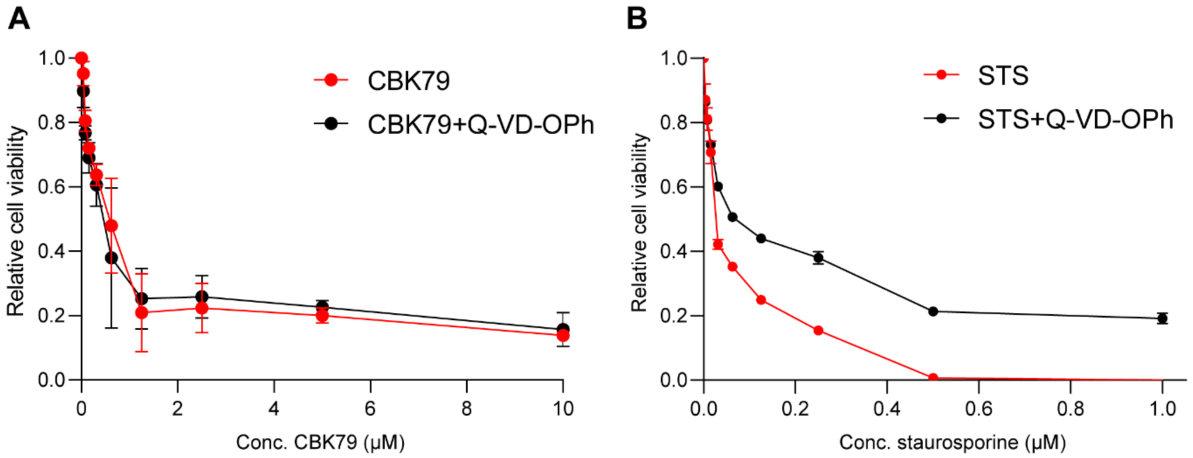

Figure S2. CBK79-mediated cell death is caspase independent. (**A**) Dose-response curves of MelJuSo Ub-YFP cells treated with CBK79 with or without co-treatment with the pan-caspase inhibitor Q-VD-OPh (20 μM) for 48 h. DMSO-treated cells were used as negative control. After incubation cell viability was determined by CellTiter-Glo. Data are shown as mean ± SD from three independent experiments. (**B**) Dose-response curves of MelJuSo Ub-YFP cells treated with staurosporine with or without co-treatment with the pan-caspase inhibitor Q-VD-OPh (20 μM) for 48 h. DMSO-treated cells were used as negative control. After incubation cell viability was determined by CellTiter-Glo. Data are shown as mean ± SD from three technical replicates of a representative experiment (from two independent experiments).

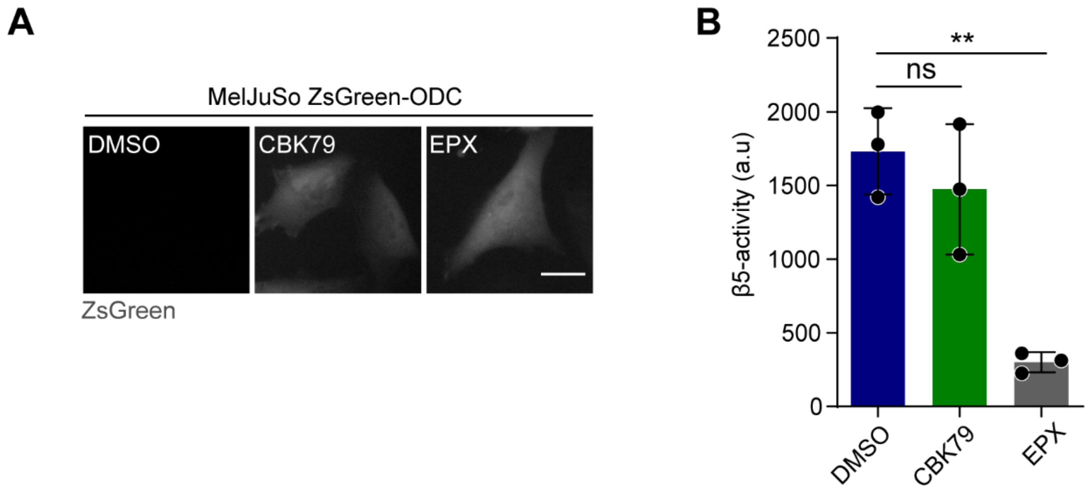

Figure S3. CBK79 impairs the degradation of a ubiquitin-independent substrate without substantially impairing the chymotrypsin activity of the proteasome. (**A**) Representative images of MelJuSo ZsGreen-ODC cells treated with the indicated compounds for 16 h. Epoxomicin (EPX, 100 nM) was used as positive control. Nuclei were counterstained with Hoechst 33342 and cells imaged in an automated widefield microscope. Scale bar: 20 µm. (**B**) MelJuSo parental cells were treated with DMSO 0.1%, CBK79 10 µM or epoxomicin (EPX, 200 nM) for 4 h. The chymotrypsin-like activity (β5-activity) of the proteasome was assessed in the corresponding lysates by following conversion of the fluorogenic Suc-LLVY-AMC substrate over 1 h. Data are shown as mean ± SD of three independent experiments. Significant differences are based on adjusted p-values (One-way ANOVA [F2,6 = 18.38, p = 0.0028] with Dunnett's multiple comparisons test). ns p = 0.5238; **p = 0.0023. Suc-LLVY-AMC = Suc-Leu-Leu-Val-Tyr-7-amido-4-methylcoumarin.

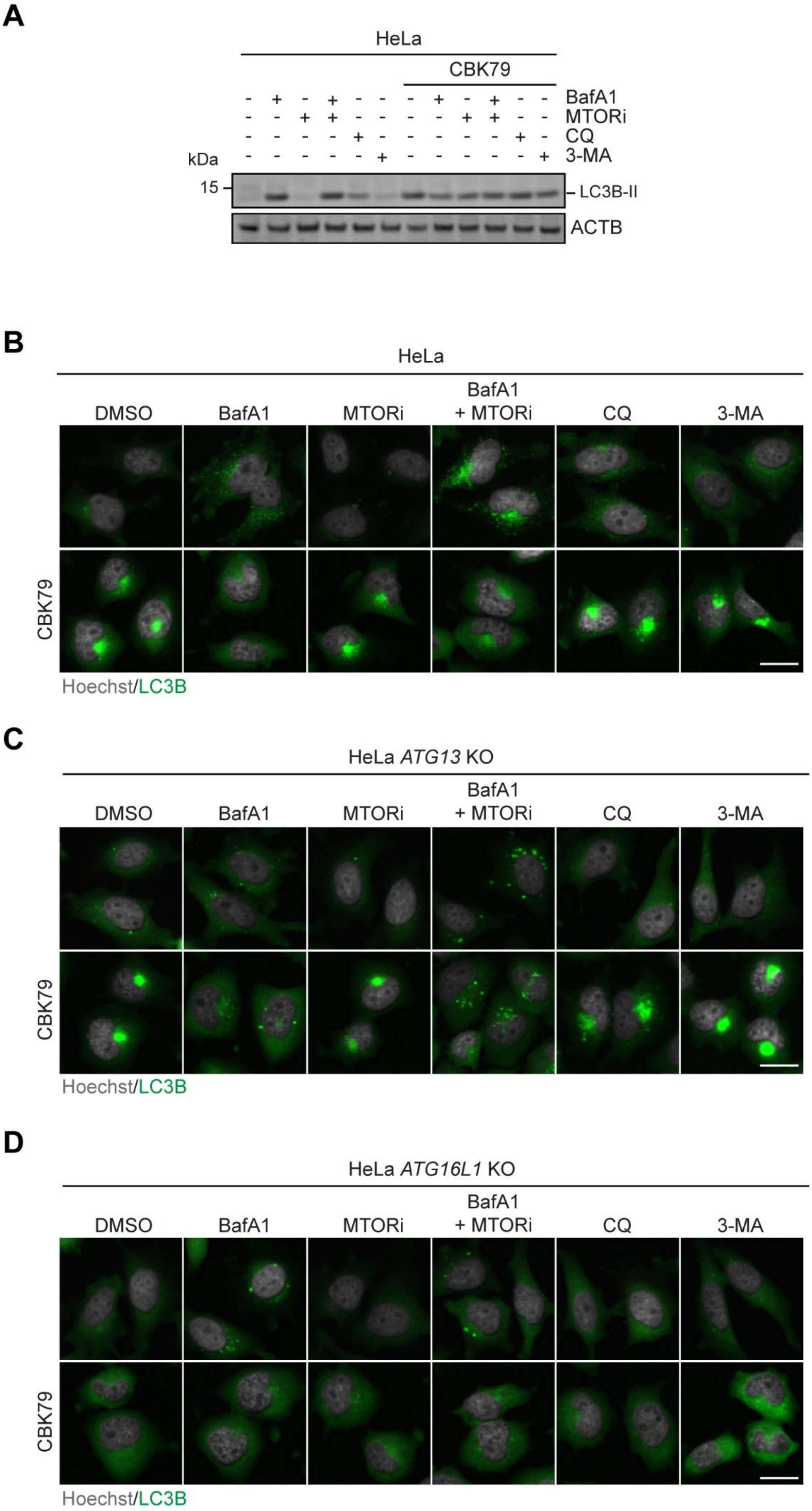
A
HeLa
CBK79
BafA1
MTORi
CQ
3-MA
kDa
15
LC3B-II
ACTB
B
HeLa
DMSO
BafA1
MTORi
BafA1 + MTORi
CQ
3-MA
CBK79
Hoechst/LC3B
C
HeLa ATG13 KO
DMSO
BafA1
MTORi
BafA1 + MTORi
CQ
3-MA
CBK79
Hoechst/LC3B
D
HeLa ATG16L1 KO
DMSO
BafA1
MTORi
BafA1 + MTORi
CQ
3-MA
CBK79
Hoechst/LC3B

Figure S4. CBK79 induces a form of non-canonical LC3B lipidation and LC3B puncta that still depends on the ATG12–ATG5-ATG16L1 conjugation complex. (**A**) HeLa cells were treated with either DMSO 0.1% or CBK79 (10 µM) in co-treatment with the indicated autophagy modulators for 4 h. Cell lysates were analyzed by immunoblotting with the indicated antibodies. Representative blots from one of three independent experiments are shown. (**B**) Maximum intensity projections of parental HeLa cells treated with the indicated compounds for 4 h before fixation and immunostaining for LC3B. Nuclei were counterstained with Hoechst 33342. Scale bar: 20 µm. (**C**) Maximum intensity projections of HeLa *ATG13* knockout (KO) cells treated with the indicated compounds for 4 h before fixation and immunostaining for LC3B. Nuclei were counterstained with Hoechst 33342. Scale bar: 20 µm. (**D**) Maximum intensity projections of HeLa *ATG16L1α* and *ATG16L1β* knockout (KO) cells treated with the indicated compounds for 4 h before fixation and immunostaining for LC3B. Nuclei were counterstained with Hoechst 33342. MTORi = MTOR inhibitor (Torin1); CQ = chloroquine; 3-MA: 3-methyladenine. Scale bar: 20 µm.

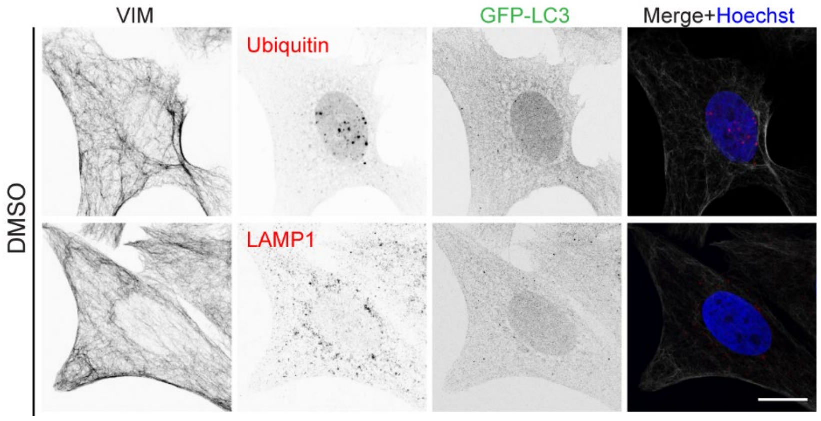

Figure S5. Control stainings for VIM, ubiquitin and LAMP1. Representative images of HOS GFP-LC3B cells treated with DMSO 0.1% for 4 h. Cells were fixed and immunostained using antibodies against VIM, ubiquitin or LAMP1. Scale bar: 20 μm.

Table S1. Chemical structures and dose-response curves of the 27 potential hits in the primary screen.

Structure	Compound ID (ChemBridge_CBCS)	Dose-response (relative YFP levels; relative cell count)
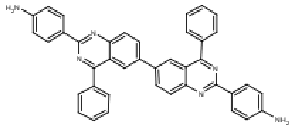	5180325_CBK261152	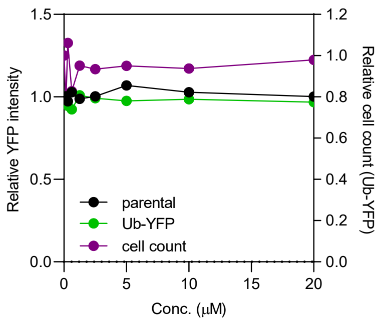
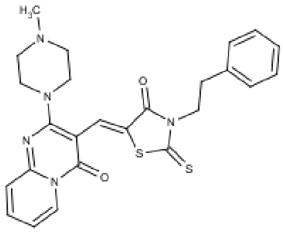	5867977_CBK263131	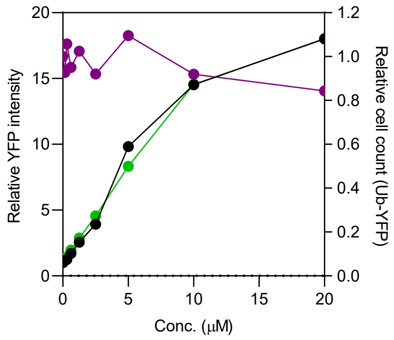
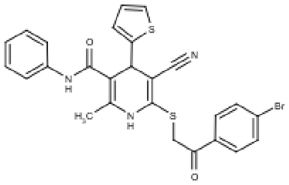	5231491_CBK261384	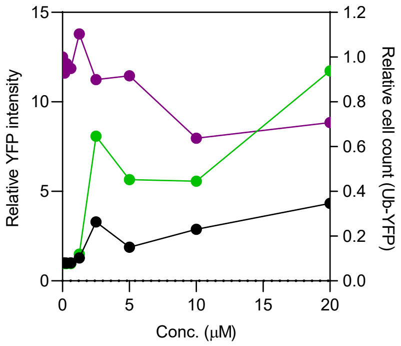
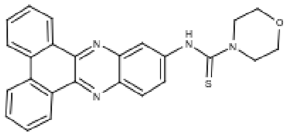	5878176_CBK263153	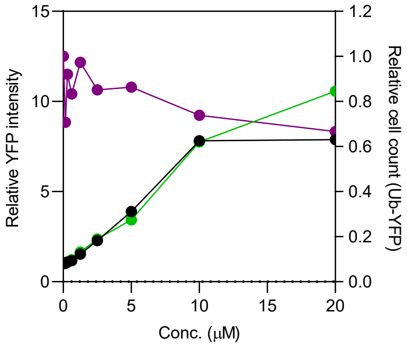

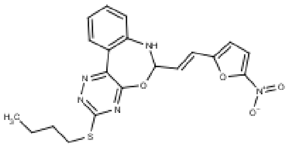	5676202_CBK262565	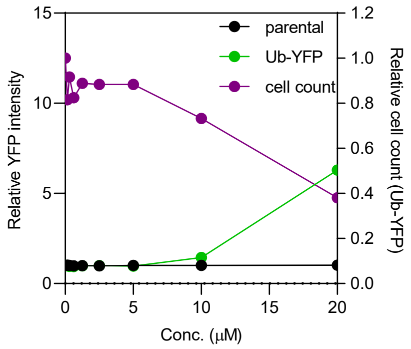
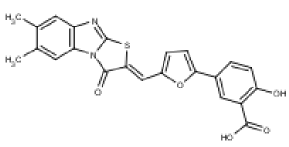	5670901_CBK262538	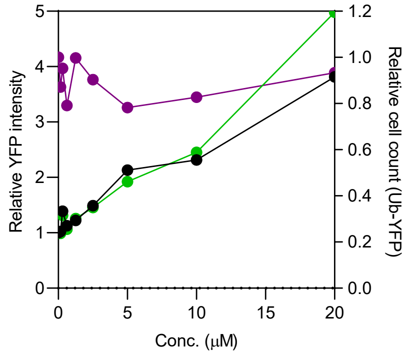
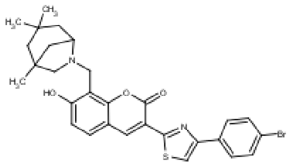	6238846_CBK263831	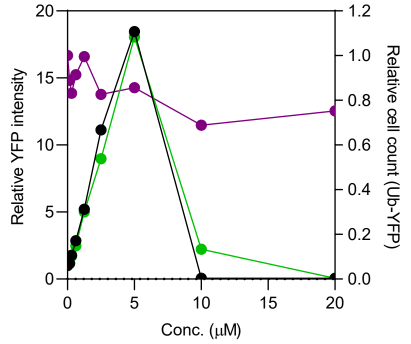
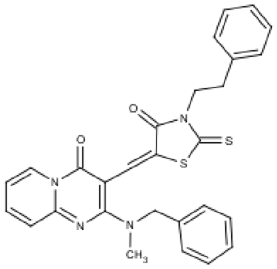	6240750_CBK263839	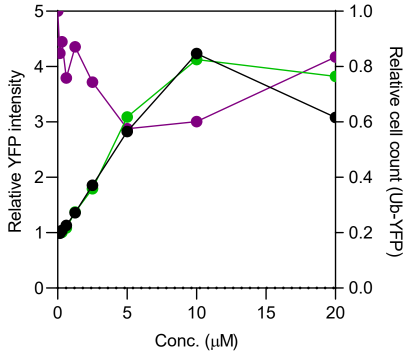

<table>
<tr>
<td>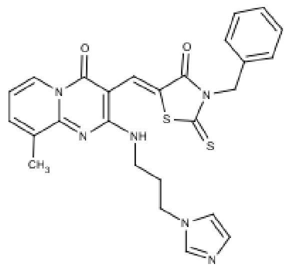</td>
<td>6239736_CBK263833</td>
<td>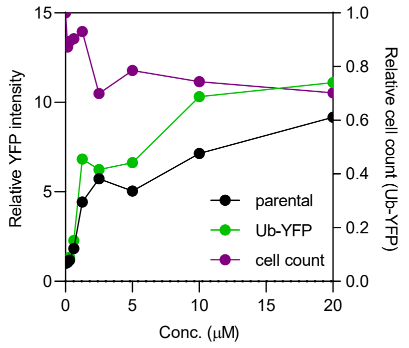
</td>
</tr>
<tr>
<td>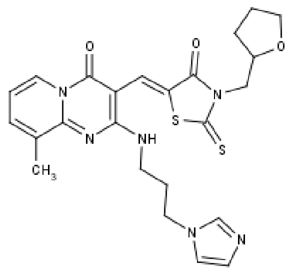</td>
<td>6237902_CBK263825</td>
<td>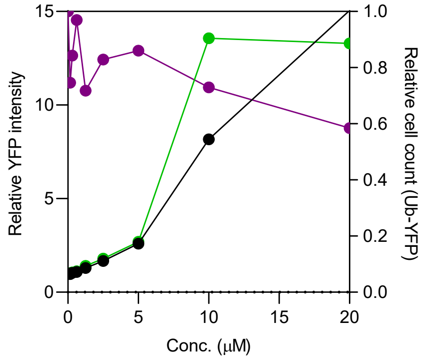
</td>
</tr>
<tr>
<td>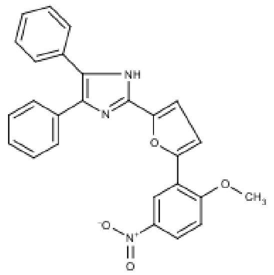</td>
<td>5663131_CBK262497</td>
<td>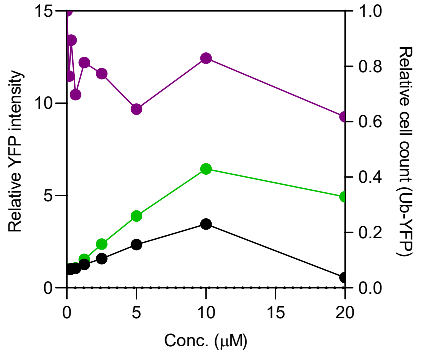
</td>
</tr>
<tr>
<td>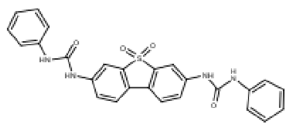</td>
<td>5584887_CBK262320</td>
<td>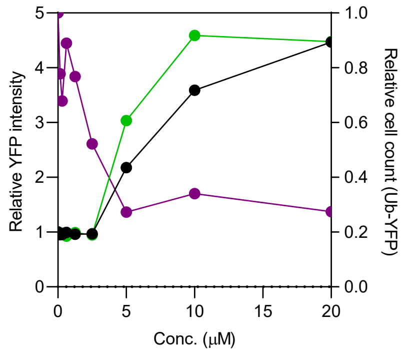
</td>
</tr>
</table>

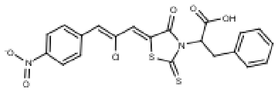	6050076_CBK263523	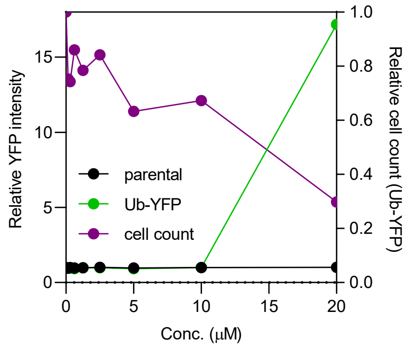
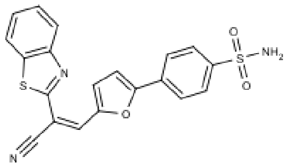	6647640_CBK264480	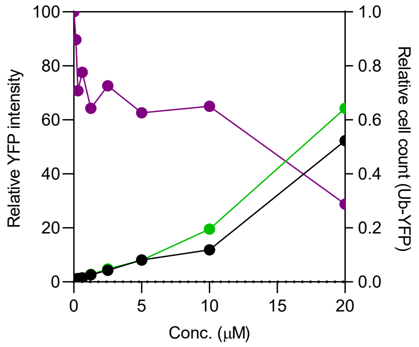
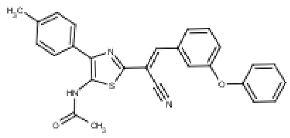	6907368_CBK264895	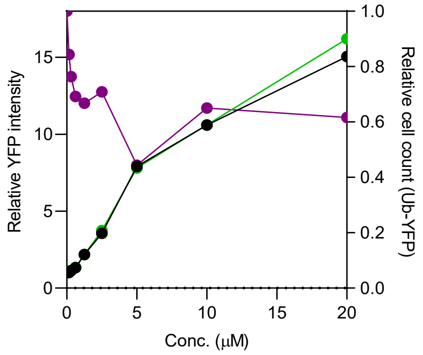
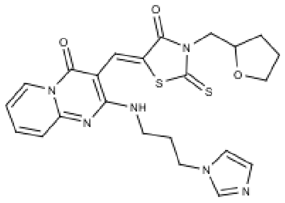	6755621_CBK111310	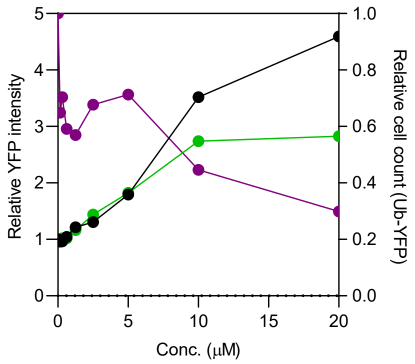

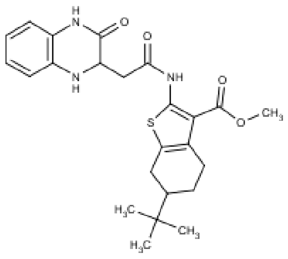	7385208_CBK265614	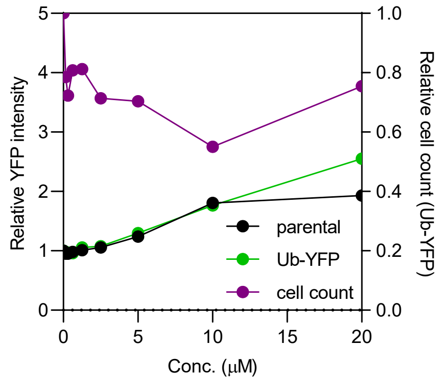
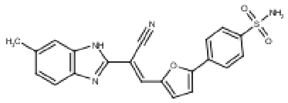	7703348_CBK266386	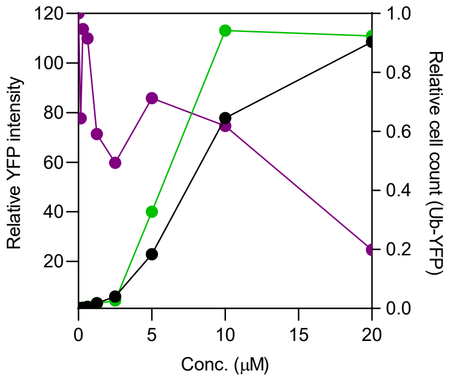
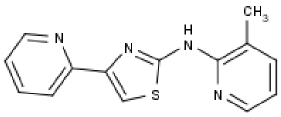	7869981_CBK267272	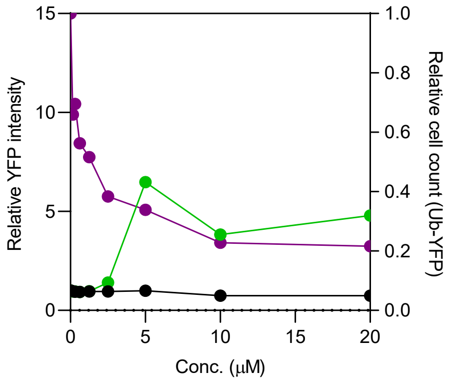
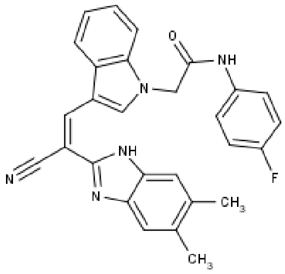	7991056_CBK269142	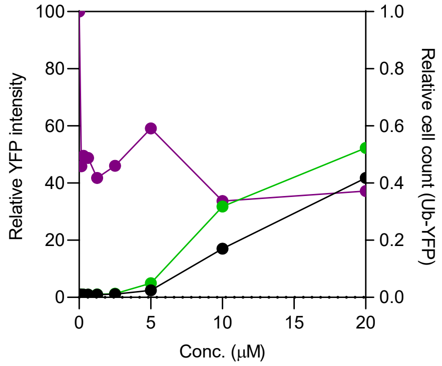

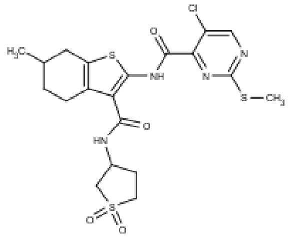	7981254_CBK268886	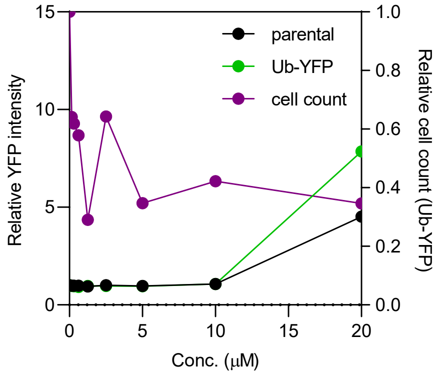
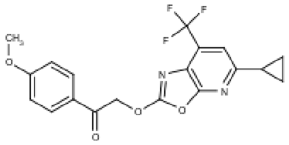	9004941_CBK269498	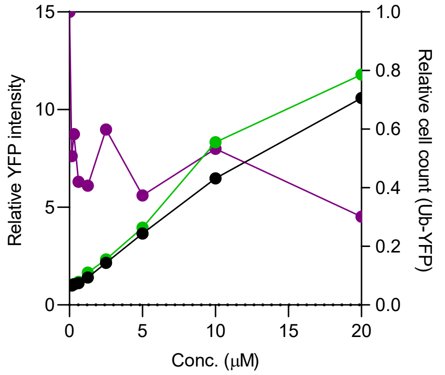
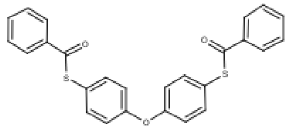	5128401_CBK270444	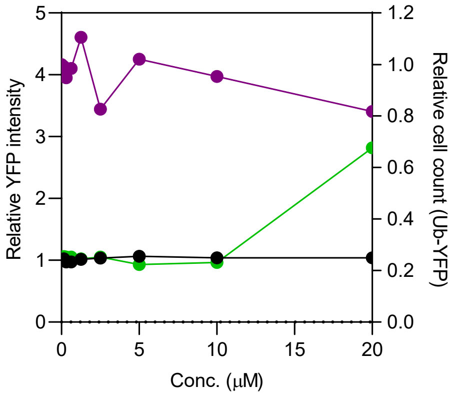
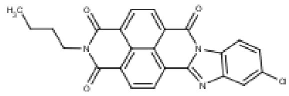	5401651_CBK272496	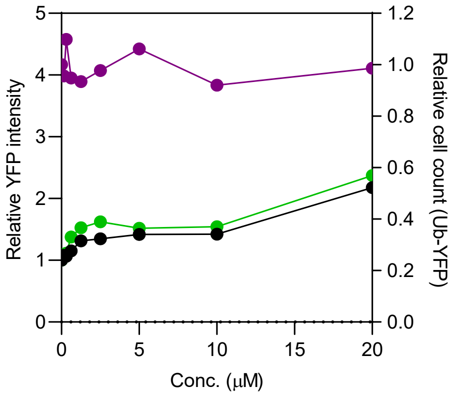

<table>
<tr>
<td>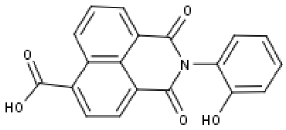</td>
<td>5405898_CBK272552</td>
<td>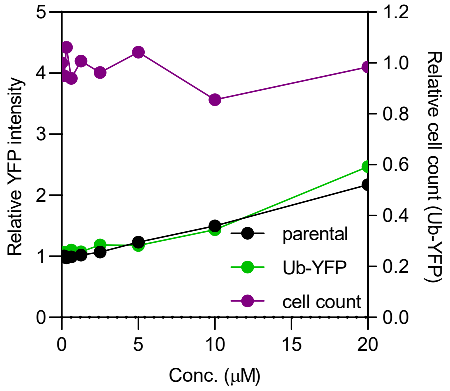
</td>
</tr>
<tr>
<td>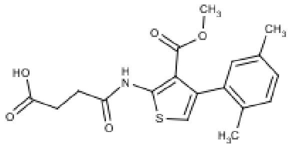</td>
<td>7281856_CBK275968</td>
<td>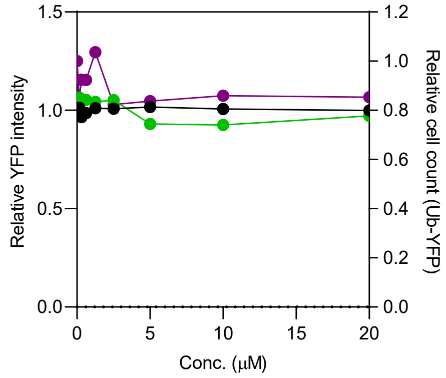
</td>
</tr>
<tr>
<td>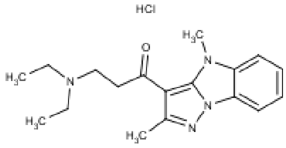</td>
<td>7907803_CBK276623C</td>
<td>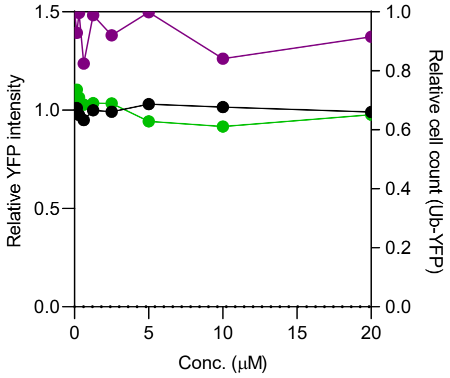
</td>
</tr>
</table>

Compound IDs are shown as identified in the ChemBridge library, as well as at the compound center at CBCS (CBK number). Compounds were tested in a dose-response experiment, ranging from 20 µM down to 0.15 µM for 20 h. DMSO at 0.2% was used as control. Nuclei were stained with Hoechst 33342 and cells were directly imaged live using the automated widefield microscope ArrayScan VTi coupled to the Cellomics software. The compounds were tested in the Ub-YFP expressing cell line as well as the parental MelJuSo cell line to exclude autofluorescent compounds. The number of cells per well (cell count) in the Ub-YFP cell line was used to identify potentially cytotoxic compounds. Data are represented as the mean YFP intensity per cell, or the cell count, relative to the DMSO control.

Table S2. Structure-activity relationships (SAR) study to understand the chemical space around the screen hit CBK267272.

Structure	ID	Mean % YFP-positive cells (10 μM)	Mean % YFP-positive cells (20 μM)	Mean YFP-intensity per cell (10 μM)	Mean YFP-intensity per cell (20 μM)	Mean EC_{50} (μM)
CBCS compounds						
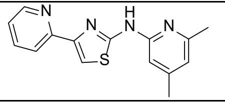	CBK288666	0.32 ± 0.36	0.12 ± 0.09	917.7 ± 18.5	896.9 ± 31.4	>20
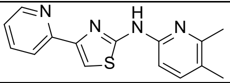	CBK288667	0.17 ±0.13	0.08 ± 0.09	896 ± 34.1	897.3 ± 30.6	>20
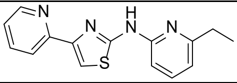	CBK288668	0.19 ± 0.22	0	883.2 ± 51.5	869.4 ± 60.8	>20
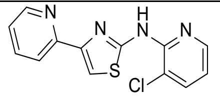	CBK288669	4.63 ± 7.71	25.14 ± 28.8	928.3 ± 144.5	1076.1 ± 366.3	NA
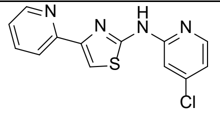	CBK288670	7.10 ± 10.95	14.83 ± 21	943 ± 158.6	1014.1 ± 276.3	11.20 ± 1.8
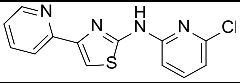	CBK288671	0.03 ± 0.07	0.2 ± 0.15	857.1 ± 59.8	859.3 ± 49.5	>20
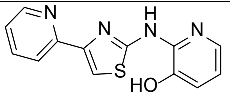	CBK288672	7.50 ± 6.36	39.6 ± 21	875.5 ± 79.4	1091.6 ± 279.5	NA
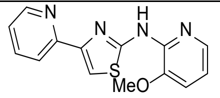	CBK288673	0.51 ± 0.34	0.35 ± 0.15	913 ± 46.8	903.6 ± 31.6	>20
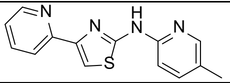	CBK288674	0.85 ± 0.46	1.2 ± 1.18	922.4 ± 69.1	901.9 ± 74.1	>20
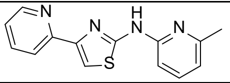	CBK288675	0.37 ± 0.37	0.2 ± 0.17	900.8 ± 76.8	890.6 ± 72.6	>20
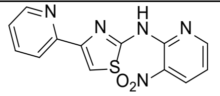	CBK288676	0.60 ± 0.47	1.44 ± 0.94	890 ± 35.8	846.4 ± 55.4	>20
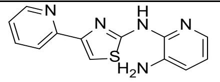	CBK288677	22.21 ± 19.26	48.15 ± 25.71	1042.6 ± 207	1229.1 ± 302.3	NA
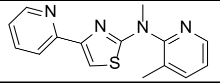	CBK288678	0.37 ± 0.26	0.5 ± 0.19	890.1 ± 55.3	881.2 ± 62.5	>20
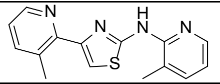	CBK288679	74.07 ± 20.65	80.34 ± 13.32	1381.5 ± 459. 5	1489.3 ± 328	2.8 ± 1.11
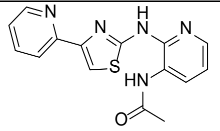	CBK288680	33.9 ± 29	62.9 ± 22.67	1162.5 ± 398. 7	1368.9 ± 427.4	3.52 ± 1.36
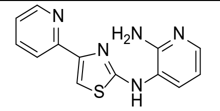	CBK288681	0.11 ± 0.13	0.42 ± 0.3	872 ± 62.6	873.7 ± 52	>20
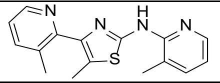	CBK291478	2.39 ± 2.18	1.96 ± 1.7	NA	NA	>20
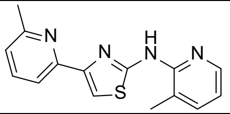	CBK291479	1.40 ± 1.13	31.27 ± 35.59	NA	NA	>20

ChemBridge compounds						
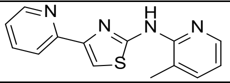	CBK267272	13	29.70	941.3 ± 180.4	1120.2	8.37
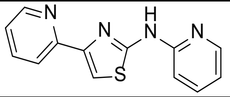	CBK281261	0.40 ± 0.16	3.3 ± 3.31	913 ± 58.3	937.4 ± 65.9	>20
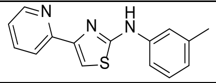	CBK281262	0.32%± 0.30	0.51 ± 0.13	915.4 ± 52.3	924 ± 82.1	>20
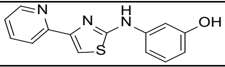	CBK281263	0.44 ± 0.44	0.32 +/ 0.31	927.9 ± 14.3	892.8 ± 78.5	>20
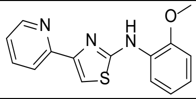	CBK281264	0.55 ± 0.09	0.68 ± 0.07	929.9 ± 21.6	906.6 ± 43.3	>20
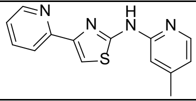	CBK281265	9.72 ± 6.96	37.3 ± 20.66	951 ± 95.4	1135.7 ± 248.1	11.27 ± 3.7
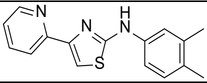	CBK281266	0	0.1 ± 0.12	833.3 ±121	876.5 ± 56	>20
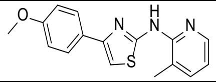	CBK281267	0.35 ± 0.22	0.31 ± 0.26	868.5 ± 69.2	875.9 ± 61.7	>20
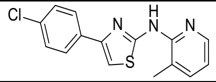	CBK281268	0.11 ± 0.14	0.11 ± 0.08	871 ± 49	880.2 ± 41.2	>20
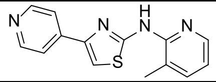	CBK281269	0.36 ± 0.18	0.35 ± 0.09	864.9 ± 57.7	871.4 ± 58	>20
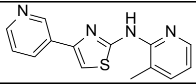	CBK281270	0.36 ± 0.35	0.31 ± 0.39	856.9 ± 65.2	833.9 ± 49.4	>20
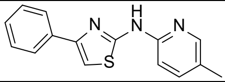	CBK281271	0.47 ± 0.32	0.41 ± 0.06	913.4 ± 52.3	916.2 ± 40.3	>20
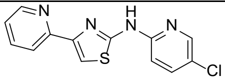	CBK281272	15.54 ± 20.77	30.25 ± 24.69	1057.3 ± 238	1158.1 ± 347.5	10.91 ± 2.52
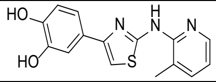	CBK281273	0.7 ± 0.36	0.17 ± 0.25	934.7 ± 22.3	891.3 ± 67.4	>20
Negative control	DMSO	0.5 ± 0.28		926.8 ± 57.4		NA
Positive control	Epoxomicin	89.27 ± 3.64		1796.2 ± 241.9		NA

The compounds studied were either synthesized (*CBCS compounds*) or purchased from ChemBridge (*Chembridge compounds*). Compound IDs are shown as identified in the compound center at CBCS. Cells were treated at 10 or 20 μM for 6 h. Nuclei were stained with Hoechst 33342 and cells were directly imaged live using the automated widefield microscope ImageXpress coupled to the MetaXpress software. The mean percentage (%) of YFP-positive cells and the mean YFP-intensity per cell (arbitrary units) ± SD from three independent experiments are shown. Cells highlighted in green correspond to mean fluorescence intensities higher than the threshold to be considered active (set as the mean fluorescence intensity of the positive control –

3*SD). The mean half-maximal effective concentration (EC_{50}) from three independent dose-response experiments (tested at 20-10-7.5-5-2.5-1.25-0.625-0.315 µM and DMSO 0.2% as control) for 6 h is shown ± SD; except for CBK267272, for which only data from one experiment converged into the non-linear fitting model. Ultimately, compounds with an EC_{50} higher than 20 µM were considered inactive. NA= non-available.

Supplementary Materials and Methods

Synthetic procedures for chemical compounds.

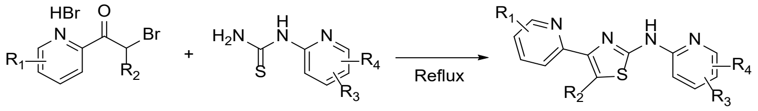

Scheme 1. Reagents and conditions: absolute ethanol, reflux, 0.5-2 h.

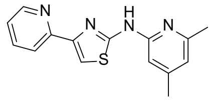

***N*-(4,6-Dimethylpyridin-2-yl)-4-(pyridin-2-yl)thiazol-2-amine** (CBK288666). A reaction mixture of 2-bromo-1-(pyridin-2-yl)ethanone hydrobromide (84.5 mg, 0.3 mmol) and 4,6-dimethylpyridin-2-ylthiourea (54.4 mg, 0.3 mmol) was dissolved in dry ethanol (2 ml) and stirred to reflux for 2 h. LC/MS indicated that all of the starting materials were consumed. One ml of water was added to the mixture, cooled to 0°C, the precipitating solid was collected by filtration, washed with ice water three times, and dried in vacuum overnight to give 70.0 mg (83%) of solid. ^{1}H NMR (DMSO$_{d6}$): 2.25 (s, 3H), 2.43 (s, 3H), 6.65 (s. 1H), 6.70 (s, 1H), 7.28-7.32 (m, 1H), 7.63 (s, 1H), 7.86 (td, *J*=7.6Hz, *J*=1.8Hz, 1H), 7.95 (d, *J*=7.6Hz, 1H), 8.58 (d, *J*=4.6Hz, 1H), 11.30 (s, 1H). ^{13}C NMR (DMSO$_{d6}$): 20.67, 23.22, 107.49, 109.64, 116.45, 119.79, 122.46, 137.14, 148.73, 149.40, 151.23, 152.55, 154.77, 160.04. LC-MS: calculated for $C_{15}H_{15}N_4S$: [M + H]$^+$, 283.10; found, 283.1.

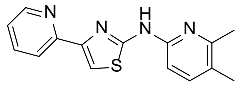

***N*-(5,6-Dimethylpyridin-2-yl)-4-(pyridin-2-yl)thiazol-2-amine** (CBK288667). A reaction mixture of 2-bromo-1-(pyridin-2-yl)ethanone hydrobromide (84.5 mg, 0.3 mmol) and 5,6-dimethylpyridin-2-ylthiourea (54.4 mg, 0.3 mmol) was dissolved in dry ethanol (2 ml) and stirred to reflux for 2 h. LC/MS indicated that all of the starting materials were consumed. One ml of water was added to the mixture, cooled to 0°C, the precipitating solid was collected by filtration, washed with ice water three times and dried in vacuum over night to give 65.0 mg (77%) of solid. ^{1}H NMR (DMSO$_{d6}$): 2.19 (s, 3H), 2.44 (s, 3H), 6.84 (d, *J*=8.2Hz, 1H), 7.30 (ddd, *J*=7.4Hz, *J*=4.8Hz, *J*=1.2Hz, 1H), 7.46 (d, *J*=8.2Hz, 1H), 7.61 (s, 1H), 7.87 (td, *J*=7.8Hz, *J*=1.8Hz, 1H), 7.95 (d, *J*=7.8Hz,

1H), 8.57-8.59 (m, 1H), 11.24 (s, 1H). ^{13}C NMR ($DMSO_{d6}$): 17.55, 21.51, 107.66, 109.25, 119.80, 122.45, 122.56, 137.15, 139.34, 148.72, 149.04, 149.38, 152.57, 153.14, 160.15. LC-MS: calculated for $C_{15}H_{15}N_4S$: $[M + H]^+$, 283.10; found, 283.1.

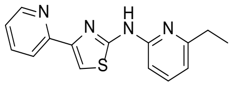

***N*-(6-Ethylpyridin-2-yl)-4-(pyridin-2-yl)thiazol-2-amine** (CBK288668). A reaction mixture of 2-bromo-1-(pyridin-2-yl)ethanone hydrobromide (84.5 mg, 0.3 mmol) and 6-ethylpyridin-2-ylthiourea (54.5 mg, 0.3 mmol) was dissolved in dry ethanol (2 ml) and stirred to reflux for 2 h. LC/MS indicated that all of starting materials were consumed. One ml of water was added to the mixture, cooled to 0°C, the precipitating solid was collected by filtration, washed with water three times and dried under vacuum over night to give 75.0 mg (88%) of product.^{1}H NMR ($DMSO_{d6}$): 1.34 (t, *J*=7.6Hz, 3H), 2.76 (q, *J*=7.6Hz, 2H), 6.80 (d, *J*=7.3Hz, 1H), 6.90 (d, *J*=8.2Hz, 1H), 7.31-7.35 (m, 1H), 7.62 (t, *J*=7.8Hz, 1H), 7.68 (s, 1H), 7.90 (td, *J*=7.6Hz, *J*=1.6Hz, 1H), 7.98 (d, *J*=7.9Hz, 1H), 8.59 (d, *J*=4.3Hz, 1H), 11.39 (s, 1H). ^{13}C NMR ($DMSO_{d6}$): 13.54, 30.22, 107.91, 109.92, 113.94, 119.99, 122.60, 137.52, 138.30, 148.47, 149.12, 151.19, 152.25, 159.88, 160.31. LC-MS: calculated for $C_{15}H_{15}N_4S$: $[M + H]^+$, 283.10; found, 283.17.

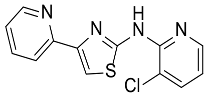

***N*-(3-Chloropyridin-2-yl)-4-(pyridin-2-yl)thiazol-2-amine** (CBK288669). A reaction mixture of 2-bromo-1-(pyridin-2-yl)ethanone hydrobromide (84.5 mg, 0.3 mmol) and 3-chloropyridin-2-ylthiourea (56.3 mg, 0.3 mmol) was dissolved in dry ethanol (2.0 ml) and stirred to reflux for 2 h. LC/MS indicated that all of starting materials were consumed. One ml of water was added to the mixture, cooled to 0°C, the precipitating solid was collected by filtration, washed with ice water three times and dried in vacuum overnight to give 75.3 mg (87%) of solid. ^{1}H NMR ($DMSO_{d6}$): 7.05 (dd, *J*=7.8Hz, *J*=4.8Hz, 1H), 7.31-7.34 (m, 1H), 7.75 (s, 1H), 7.88 (td, *J*=7.7Hz, *J*=1.7Hz, 1H), 7.94 (dd, *J*=7.8Hz, *J*=1.4Hz, 1H), 8.06 (d, *J*=7.9Hz, 1H), 8.33 (dd, *J*=4.8Hz, *J*=1.4Hz, 1H), 8.60 (d, *J*=4.5Hz, 1H), 10.71 (s, 1H). ^{13}C NMR ($DMSO_{d6}$): 110.71, 115.95, 117.41, 120.12, 122.67, 137.21, 138.21, 144.66, 147.78, 148.82, 149.41, 152.23, 159.31. LC-MS: calculated for $C_{13}H_{10}ClN_4S$: $[M + H]^+$, 289.03; found, 289.02.

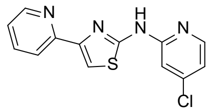

***N*-(4-Chloropyridin-2-yl)-4-(pyridin-2-yl)thiazol-2-amine** (CBK288670). A reaction mixture of 2-bromo-1-(pyridin-2-yl)ethanone hydrobromide (84.5 mg, 0.3 mmol) and 4-chloropyridin-2-ylthiourea (56.3 mg, 0.3 mmol) was dissolved in dry ethanol (4.0 ml) and stirred to reflux for 2 h. LC/MS indicated that all of the starting materials were consumed. One ml of water was added to the mixture, cooled to 0°C, the precipitating solid was collected by filtration, washed with ice water three times and dried in vacuum over night to give 72.1 mg (83%) of solid. ^{1}H NMR (DMSO$_{d6}$): 7.05 (dd, *J*=5.6Hz, *J*=1.8Hz, 1H), 7.17 (d, *J*=1.7Hz, 1H), 7.32 (ddd, *J*=7.4Hz, *J*=4.8Hz, *J*=1.2Hz, 1H), 7.70 (s, 1H), 7.88 (td, *J*=7.6Hz, *J*=1.8Hz, 1H), 7.95 (d, *J*=7.9Hz, 1H), 8.31 (d, *J*=5.6Hz, 1H), 8.59 (ddd, *J*=4.8Hz, *J*=1.6Hz, *J*=0.9Hz, 1H), 11.63 (s, 1H). ^{13}C NMR (DMSO$_{d6}$): 109.97, 110.18, 116.19, 119.86, 122.64, 137.22, 143.53, 148.26, 149.02, 149.48, 152.29, 152.92, 159.46. LC-MS: calculated for $C_{13}H_{10}ClN_4S$: [M + H]$^+$, 289.03; found, 288.95.

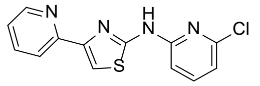

***N*-(6-Chloropyridin-2-yl)-4-(pyridin-2-yl)thiazol-2-amine** (CBK288671). A reaction mixture of 2-bromo-1-(pyridin-2-yl)ethanone hydrobromide (84.5 mg, 0.3 mmol) and 6-chloropyridin-2-ylthiourea (56.3 mg, 0.3 mmol) was dissolved in dry ethanol (3 ml) and stirred to reflux for 2 h. LC/MS indicated that all of starting materials were consumed. 1ml of water was added to the mixture, cooled to 0°C, the precipitating solid was collected by filtration, washed with ice water three times and purified by preparative HPLC (acetonitrile/water=20% to 100%/25min, flow rate=20 mL/min, with 0.005% formic acid, wavelength=214nm) to give 65 mg (75%) of pure product. ^{1}H NMR (DMSO$_{d6}$): 7.03 (d, *J*=7.6Hz, 1H), 7.10 (d, *J*=8.1Hz, 1H), 7.32 (ddd, *J*=7.4Hz, *J*=4.8Hz, *J*=1.2Hz, 1H), 7.74 (s, 1H), 7.77 (t, *J*=7.9Hz, 1H), 7.88 (td, *J*=7.6Hz, *J*=1.8Hz, 1H), 7.96 (d, *J*=7.9Hz, 1H), 8.60 (ddd, *J*=4.7Hz, *J*=1.6Hz, *J*=0.9Hz, 1H), 11.74 (s, 1H). ^{13}C NMR (DMSO$_{d6}$): 109.37, 110.28, 115.26, 119.90, 122.67, 137.24, 140.97, 147.08, 149.06, 149.48, 151.89, 152.27, 159.36. LC-MS: calculated for $C_{13}H_{10}ClN_4S$: [M + H]$^+$, 289.03; found, 289.02.

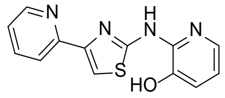

2-((4-(Pyridin-2-yl)thiazol-2-yl)amino)pyridin-3-ol (CBK288672). A reaction mixture of 2-bromo-1-(pyridin-2-yl)ethanone hydrobromide (84.5 mg, 0.3 mmol) and 3-hydroxylpyridin-2-ylthiourea (61.7 mg, 0.3 mmol) was dissolved in dry ethanol (2 ml) and stirred to reflux for 15 min. LC/MS indicated that all of starting materials were consumed. One ml of water was added to the mixture, cooled to 0°C, the precipitating solid was collected, washed with water three times, purified with preparative HPLC (acetonitrile/water=10% to 80%/30min, flow rate=20mL/min, with 0.005% formic acid, wavelength=310nm) to give 52 mg (64%) of product after lyophilization. ^{1}H NMR ($DMSO_{d6}$): 6.86 (dd, *J*=7.7Hz, *J*=5.0Hz, 1H), 7.14 (dd, *J*=7.8Hz, *J*=1.4Hz, 1H), 7.31 (ddd, *J*=7.5Hz, *J*=4.8Hz, *J*=1.0Hz, 1H), 7.66 (s, 1H), 7.82 (dd, *J*=4.9Hz, *J*=1.3Hz, 1H), 7.88 (td, *J*=7.7Hz, *J*=1.8Hz, 1H), 8.01 (d, *J*=7.9Hz, 1H), 8.59 (d, *J*=4.1Hz, 1H), 9.88 (s, 1H), 10.42 (s, 1H). ^{13}C NMR ($DMSO_{d6}$): 109.65, 116.82, 120.10, 120.35, 122.57, 135.99, 137.18, 140.43, 141.83, 148.74, 149.34, 152.37, 159.48. LC-MS: calculated for $C_{13}H_{11}N_4OS$: $[M + H]^+$, 271.07; found, 271.03.

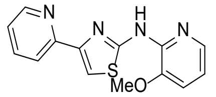

***N*-(3-Methoxypyridin-2-yl)-4-(pyridin-2-yl)thiazol-2-amine** (CBK288673). A reaction mixture of 2-bromo-1-(pyridin-2-yl)ethanone hydrobromide (84.5 mg, 0.3 mmol) and 3-methoxypyridin-2-ylthiourea (55.0 mg, 0.3 mmol) was dissolved in dry ethanol (2 ml) and stirred to reflux for 2 h. LC/MS indicated that all of starting materials were consumed. One ml of water was added to the mixture, cooled to 0°C, the precipitating solid was collected by filtration, washed with ice water three times and dried in vacuum over night to give 70.0 mg (82%) of solid. ^{1}H NMR ($DMSO_{d6}$): 3.90 (s, 3H), 6.97 (dd, *J*=7.9Hz, *J*=5.0Hz, 1H), 7.31 (ddd, *J*=7.5Hz, *J*=4.8Hz, *J*=1.1Hz, 1H), 7.36 (dd, *J*=7.9Hz, *J*=1.2Hz, 1H), 7.68 (s, 1H), 7.87 (td, *J*=7.8Hz, *J*=1.8Hz, 1H), 7.91 (dd, *J*=5.0Hz, *J*=1.3Hz, 1H), 8.02 (d, *J*=7.9Hz, 1H), 8.59 (ddd, *J*=4.8Hz, *J*=1.7Hz, *J*=0.8Hz, 1H), 10.21 (s, 1H). ^{13}C NMR ($DMSO_{d6}$): 55.82, 109.85, 116.48, 116.97, 120.06, 122.56, 136.81, 137.12, 142.29, 142.61, 148.88, 149.40, 152.43, 159.33. LC-MS: calculated for $C_{14}H_{13}N_4OS$: $[M + H]^+$, 285.08; found, 285.1.

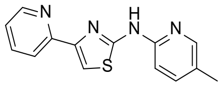

N-(5-Methylpyridin-2-yl)-4-(pyridin-2-yl)thiazol-2-amine (CBK288674). A reaction mixture of 2-bromo-1-(pyridin-2-yl)ethanone hydrobromide (84.5 mg, 0.3 mmol) and 5-methylpyridin-2-ylthiourea (50.2 mg, 0.3 mmol) was dissolved in dry ethanol (4 ml) and stirred to reflux for 2 h. LC/MS indicated that all of starting materials were consumed. One ml of water was added to the mixture, cooled to 0°C, the precipitating solid was collected by filtration, washed with ice water three times and dried in vacuum over night to give 58.1 mg (72%) of solid. ^{1}H NMR (DMSO$_{d6}$): 2.23 (s, 3H), 7.03 (d, *J*=8.4Hz, 1H), 7.30 (ddd, *J*=7.4Hz, *J*=4.8Hz, *J*=1.0Hz, 1H), 7.56 (dd, *J*=8.4Hz, *J*=2.1Hz, 1H), 7.61 (s, 1H), 7.87 (td, *J*=7.6Hz, *J*=1.7Hz, 1H), 7.95 (d, *J*=7.9Hz, 1H), 8.14 (s, 1H), 8.58 (d, *J*=4.1Hz, 1H), 11.32 (s, 1H). ^{13}C NMR (DMSO$_{d6}$): 17.19, 109.19, 110.39, 119.84, 122.49, 124.77, 137.15, 138.83, 145.79, 148.87, 149.42, 149.85, 152.51, 160.00. LC-MS: calculated for $C_{14}H_{13}N_4S$: [M + H]$^+$, 269.09; found, 269.08.

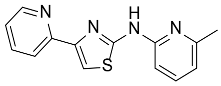

N-(6-Methylpyridin-2-yl)-4-(pyridin-2-yl)thiazol-2-amine (CBK288675). A reaction mixture of 2-bromo-1-(pyridin-2-yl)ethanone hydrobromide (84.5 mg, 0.3 mmol) and 6-methylpyridin-2-ylthiourea (50.2 mg, 0.3 mmol) was dissolved in dry ethanol (3 ml) and stirred to reflux for 2 h. LC/MS indicated that all of starting materials were consumed. One ml of water was added to the mixture, cooled to 0°C, the precipitating solid was collected by filtration, washed with ice water three times and dried in vacuum over night to give 54.5 mg (68%) of solid. ^{1}H NMR (DMSO$_{d6}$): 2.47 (s, 3H), 6.79 (d, *J*=7.4Hz, 1H), 6.90 (d, *J*=8.2Hz, 1H), 7.30 (ddd, *J*=7.4Hz, *J*=4.8Hz, *J*=1.2Hz, 1H), 7.60 (dd, *J*=8.0Hz, *J*=7.6Hz, 1H), 7.65 (s, 1H), 7.87 (td, *J*=7.6Hz, *J*=1.8Hz, 1H), 7.96 (d, *J*=7.9Hz, 1H), 8.59 (ddd, *J*=4.7Hz, *J*=1.7Hz, *J*=0.8Hz, 1H), 11.38 (s, 1H). ^{13}C NMR (DMSO$_{d6}$): 23.40, 107.53, 109.64, 114.97, 119.81, 122.48, 137.15, 138.20, 148.76, 149.40, 151.09, 152.53, 155.14, 159.87. LC-MS: calculated for $C_{14}H_{13}N_4S$: [M + H]$^+$, 269.09; found, 269.02.

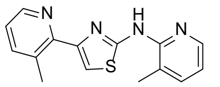

***N*,4-Bis(3-methylpyridin-2-yl)thiazol-2-amine** (CBK288679). A reaction mixture of 2-bromo-1-(3-methylpyridin-2-yl)ethanone hydrobromide (1032 mg, 3.5 mmol) and 3-methylpyridin-2-ylthiourea (585.8 mg, 3.5 mmol) was dissolved in dry ethanol (14 mL) and stirred to reflux for 0.5 h. Water (1.5 mL) was added to the mixture, basified to pH=9 with NaOH solution (5%), cooled to 0°C, precipitating product was collected by filtration, washed with water and dried under vacuum over night to give 825 mg (83.5%) of product. ^{1}H NMR (DMSO$_{d6}$): 2.36 (s, 3H), 2.59 (s, 3H), 6.89-6.92 (m, 1H), 7.24-7.27 (m, 1H), 7.36 (s, 1H), 7.56 (d, *J*=7.2Hz, 1H), 7.67 (d, *J*=7.5Hz, 1H), 8.19 (d, *J*=4.1Hz, 1H), 8.43 (d, *J*=4.0Hz, 1H), 10.32 (s, 1H); ^{13}C NMR (DMSO$_{d6}$): 16.94, 20.18, 111.61, 116.23, 119.20, 122.42, 130.98, 138.50, 138.96, 143.37, 146.43, 149.64, 150.14, 152.09, 158.86. LC-MS: calculated for $C_{15}H_{15}N_4S$: [M + H]$^+$, 283.10; found, 283.1.

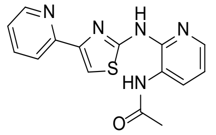

***N*-(2-((4-(Pyridin-2-yl)thiazol-2-yl)amino)pyridin-3-yl)acetamide** (CBK288680). A reaction mixture of 2-bromo-1-(pyridin-2-yl)ethanone hydrobromide (112.4 mg, 0.4 mmol) and 3-acetamidopyridin-2-ylthiourea (84.1 mg, 0.4 mmol) was dissolved in dry ethanol (2 ml) and stirred to reflux for 2 h. One ml of water was added to the mixture, cooled to 0°C, the precipitating solid was collected by filtration, washed with water three times and dried under vacuum to give 97 mg (78%) of solid.^{1}H NMR (DMSO$_{d6}$): 2.16 (s, 3H), 7.01 (dd, *J*=7.8Hz, *J*=4.9Hz, 1H), 7.32 (ddd, *J*=7.4Hz, *J*=4.8Hz, *J*=1.0Hz, 1H), 7.69 (s, 1H), 7.88 (td, *J*=7.8Hz, *J*=1.8Hz, 1H), 7.96 (d, *J*=7.5Hz, 1H), 8.01 (d, *J*=7.9Hz, 1H), 8.16 (dd, *J*=4.8Hz, *J*=1.4Hz, 1H), 8.60 (d, *J*=4.0Hz, 1H), 9.56 (s, 1H), 10.77 (s, 1H). ^{13}C NMR (DMSO$_{d6}$): 23.78, 110.11, 116.19, 119.94, 120.73, 122.61, 132.54, 137.26, 141.95, 144.47, 149.40, 152.36, 159.76, 169.32. LC-MS: calculated for $C_{15}H_{14}N_5OS$: [M + H]$^+$, 312.09; found, 312.10.

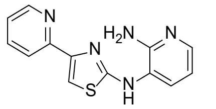

***N*³-(4-(Pyridin-2-yl)thiazol-2-yl)pyridine-2,3-diamine** (CBK288681). A reaction mixture of 2-bromo-1-(pyridin-2-yl)ethanone hydrobromide (106.2 mg, 0.38 mmol) and 2-aminopyridin-3-ylthiourea (63.5 mg, 0.38 mmol) was dissolved in dry ethanol (2.5 ml) and stirred to reflux for 30 min. LC/MS indicated that all the starting materials were

consumed. One ml of water was added to the mixture, cooled to 0°C, the precipitating product was collected by filtration, washed with water three times and dried in vacuum to give 92.6 mg (91%) of product. ^{1}H NMR (DMSO$_{d6}$): 5.87 (s, 2H), 6.65 (dd, *J*=7.7Hz, *J*=4.9Hz, 1H), 7.30 (dd, *J*=7.2Hz, *J*=4.8Hz, *J*=1.2Hz, 1H), 7.51 (s, 1H), 7.73 (dd, *J*=4.9Hz, *J*=1.5Hz, 1H), 7.86 (td, *J*=7.7Hz, *J*=1.7Hz, 1H), 7.92 (d, *J*=7.8Hz, 1H), 8.20 (dd, *J*=7.8Hz, *J*=1.4Hz, 1H), 8.57 (d, *J*=4.3Hz, 1H), 9.25 (s, 1H). ^{13}C NMR (DMSO$_{d6}$): 106.97, 112.62, 120.28, 121.90, 122.61, 127.15, 137.23, 141.92, 149.40, 150.24, 151.41, 152.12, 165.18. LC-MS: calculated for $C_{13}H_{12}N_5S$: $[M + H]^+$, 270.08; found, 270.09.

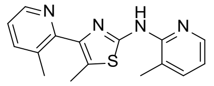

5-Methyl-*N*,4-bis(3-methylpyridin-2-yl)thiazol-2-amine (CBK291478). A reaction mixture of 2-bromo-1-(3-methylpyridin-2-yl)propan-1-one hydrobromide (92.7 mg, 0.3 mmol) and 3-methylpyridin-2-ylthiourea (50.2 mg, 0.3 mmol) was dissolved in ethanol (2 ml) and stirred to reflux for 1.5 h. LC/MS indicated that all the starting materials were consumed. The mixture was poured onto ice and extracted with ethyl acetate three times (6 ml each time) and dried over sodium sulfate. After removing the solvent, the residue was purified with flash chromatography (ethyl acetate) to give 69 mg of yellow product. ^{1}H NMR indicated that the product was not pure enough. It was purified by preparative HPLC (acetonitrile/water=20% to 100%/25min, flow rate=20 mL/min, with 0.005% formic acid, wavelength=214nm) to give 50 mg (56%) of pure product. ^{1}H NMR (CDCl$_3$): 2.28 (s, 3H), 2.32 (s, 3H), 2.33 (s, 3H), 6.84 (dd, *J*=7.2Hz, *J*=5.1Hz, 1H), 7.20 (dd, *J*=7.7Hz, *J*=4.7Hz, 1H), 7.42 (ddd, *J*=7.3Hz, *J*=1.6Hz, *J*=0.8Hz, 1H), 7.59 (ddd, *J*=7.7Hz, *J*=1.5Hz, *J*=0.7Hz, 1H), 8.23 (d, *J*=4.4Hz, 1H), 8.35 (br. 1H), 8.53 (dd, *J*=4.7Hz, *J*=1.1Hz, 1H). ^{13}C NMR (CDCl$_3$): 11.56, 16.75, 19.11, 116.55, 117.81, 122.79, 123.27, 132.92, 138.30, 138.40, 143.38, 144.44, 147.08, 149.74, 153.25, 156.94. LC-MS: calculated for $C_{16}H_{17}N_4S$: $[M + H]^+$, 297.12; found, 297.13.

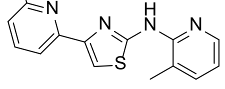

***N*-(3-methylpyridin-2-yl)-4-(6-methylpyridin-2-yl)thiazol-2-amine** (CBK291479). A reaction mixture of 2-bromo-1-(6-methylpyridin-2-yl)ethanone hydrobromide (88.5 mg, 0.3 mmol) and 3-methylpyridin-2-ylthiourea (50.2 mg, 0.3 mmol) was dissolved in dry

ethanol (2 ml) and stirred to reflux for 30 min. Upon completion of the reaction, the mixture was cooled to room temperature, poured onto ice and adjusted to pH=8.0 with sodium bicarbonate solution (10%). The precipitating product was filtered, washed with ice water three times and dried in vacuum overnight to give 72.6 mg (86%) of solid. ^{1}H NMR ($DMSO_{d6}$): 2.37 (s, 3H), 2.52 (s, 3H), 6.92 (dd, *J*=7.2Hz, *J*=5.2Hz, 1H), 7.17 (d, *J*=7.6Hz, 1H), 7.56-7.58 (m, 1H), 7.62 (s, 1H), 7.74-7.77 (m, 1H), 7.85 (d, *J*=7.6Hz, 1H), 8.19 (dd, *J*=4.8Hz, *J*=1.2Hz, 1H), 10.48 (s, 1H). ^{13}C NMR ($DMSO_{d6}$): 17.38, 24.72, 110.21, 116.83, 117.61, 119.79, 122.32, 137.74, 139.02, 143.78, 149.48, 150.52, 152.42, 158.06, 160.48. LC-MS: calculated for $C_{15}H_{15}N_4S$: $[M + H]^+$, 283.10; found, 283.04.

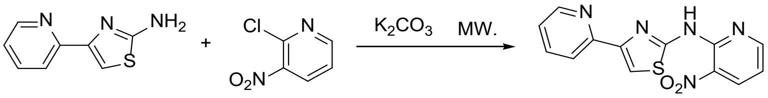

Scheme 2. Reagents and conditions: K_2CO_3, absolute ethanol, microwave 125°C, 1 h.

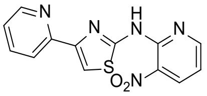

***N*-(3-Nitropyridin-2-yl)-4-(pyridin-2-yl)thiazol-2-amine** (CBK288676). To a 2-chloro-3-nitropyridine (79.3 mg, 0.5 mmol) and 4-(pyridin-2-yl)thiazol-2-amine (88.6 mg, 0.5 mmol) in absolute ethanol (5 ml) solution potassium carbonate was added. The resulting mixture was heated with microwave oven at 125°C for 1 h. A product was purified with preparative HPLC (acetonitrile/water=10% to 80%/30 min, flow rate=20 mL/min, with 0.005% formic acid) to give 13 mg (8.7%) of solid after lyophilization. ^{1}H NMR ($CDCl_3$): 7.08 (dd, *J*=8.3Hz, *J*=4.6Hz, 1H), 7.23-7.25 (m, 1H), 7.79-7.83 (m, 2H), 8.08 (d, *J*=7.9Hz, 1H), 8.63-8.65 (m, 2H), 8.69 (dd, *J*=4.6Hz, *J*=1.6Hz, 1H), 11.26 (s, 1H). ^{13}C NMR ($CDCl_3$): 112.65, 115.83, 121.04, 122.80, 129.09, 135.70, 137.29, 145.96, 149.46, 150.03, 152.56, 153.84, 157.64. LC-MS: calculated for $C_{13}H_{10}N_5O_2S$: $[M + H]^+$, 300.06; found, 299.96.

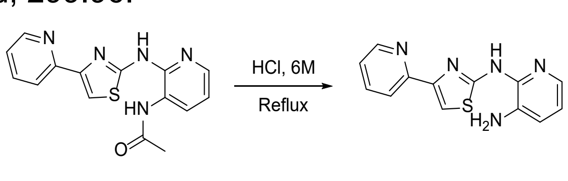

Scheme 3. Reagents and conditions: HCl (6.0 M), ethanol, reflux, 10 min.

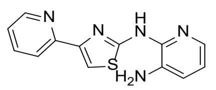

N^2-(4-(Pyridin-2-yl)thiazol-2-yl)pyridine-2,3-diamine (CBK288677). *N*-(2-((4-(Pyridin-2-yl)thiazol-2-yl)amino)pyridin-3-yl)acetamide (40.0 mg, 0.13 mmol) was suspended in ethanol (1 ml) and hydrogen chloride solution (1 ml, 6.0 M) was added. The mixture was refluxed for 10 min, then neutralized with sodium bicarbonate solution (10%) to pH=8 and extracted with ethyl acetate three times. The organic phase was removed under pressure and purified with preparative HPLC (acetonitrile/water=10% to 80%/30 min, flow rate=20 mL/min, with 0.005% formic acid) to give 28 mg (81%) of product after lyophilization. ^{1}H NMR ($DMSO_{d6}$): 5.46 (s, 2H), 6.77 (dd, *J*=7.7Hz, *J*=4.9Hz, 1H), 6.98 (dd, *J*=7.7Hz, *J*=1.5Hz, 1H), 7.30 (ddd, *J*=7.4Hz, *J*=4.8Hz, *J*=1.1Hz, 1H), 7.60 (s, 1H), 7.63 (dd, *J*=4.8Hz, *J*=1.4Hz, 1H), 7.87 (td, *J*=7.6Hz, *J*=1.8Hz, 1H), 7.99 (d, *J*=7.9Hz, 1H), 8.59 (ddd, *J*=4.7Hz, *J*=1.6Hz, *J*=0.8Hz, 1H), 10.46 (s, 1H). ^{13}C NMR ($DMSO_{d6}$): 109.34, 117.12, 119.87, 122.46, 131.54, 132.85, 137.13, 139.09, 148.75, 149.41, 152.59, 160.30. LC-MS: calculated for $C_{13}H_{12}N_5S$: $[M + H]^+$, 270.08; found, 270.02.

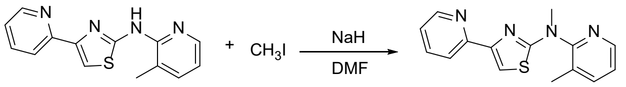

Scheme 4. Reagents and conditions: CH_3I, NaH (60%), *dimethylformamide (*DMF), RT, overnight.

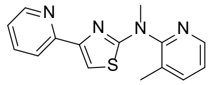

***N*-Methyl-*N*-(3-methylpyridin-2-yl)-4-(pyridin-2-yl)thiazol-2-amine** (CBK288678). To a solution of *N*-(3-methylpyridin-2-yl)-4-(pyridin-2-yl)thiazol-2-amine (60.0 mg, 0.23 mmol) in DMF (3 ml) was added sodium hydride (13.5 mg, 60%, 0.23 mmol), 2 minutes later methyl iodide (14.0 µl, 0.23 mmol) was added. The reaction mixture was stirred at room temperature overnight. The reaction was quenched with saturated ammonium chloride and extracted with ethyl acetate three times (10 ml x 3). The combined organic phases were dried over sodium sulfate, concentrated and purified with preparative HPLC (acetonitrile/water=20% to 80%/30 min, flow rate=20 mL/min, with 0.005% formic acid) to give 14.8 mg (23%) of product after lyophilization. ^{1}H NMR ($DMSO_{d6}$): 2.08 (s, 0.6H), 2.34 (s, 2.4H), 3.20 (s, 0.7H), 3.76 (s, 2.3H), 4.83 (s, 0.2H), 6.83 (dd,

J=7.2Hz, *J*=5.0Hz, 0.8H), 6.87 (dd, *J*=7.2Hz, *J*=5.0Hz, 0.2H), 6.95 (s, 0.7H), 7.16 (ddd, *J*=7.5Hz, *J*=4.8Hz, *J*=0.8Hz, 0.2H), 7.45-7.49 (m, 1H), 7.51-7.54 (m, 1H), 7.75 (d, *J*=7.9Hz, 0.8H), 7.81 (td, *J*=7.8Hz, *J*=1.8Hz, 0.2H), 7.95 (td, *J*=7.8Hz, *J*=1.8Hz, 0.8H), 8.07 (d, *J*=3.5Hz, 0.2H), 8.15-8.17 (m, 0.2H), 8.22 (dd, *J*=4.9Hz, *J*=1.4Hz, 0.7H), 8.71 (ddd, *J*=4.8Hz, *J*=1.6Hz, *J*=0.8Hz, 0.7H). ^{13}C NMR ($DMSO_{d6}$): 17.13, 17.34, 34.19, 51.18, 80.37, 106.27, 115.93, 120.36, 121.99, 123.54, 123.88, 126.56, 137.16, 137.45, 137.53, 137.72, 143.11, 143.97, 147.86, 149.16, 148.98, 156.45, 157.76, 158.64, 158.97, 161.15. LC-MS: calculated for $C_{15}H_{15}N_4S$: $[M + H]^+$, 283.10; found, 283.04.
